# Bis(di­methyl­formamide)­penta­kis­(μ-*N*,2-dioxido­benzene-1-carb­ox­imidato)tetra­kis­(1-methyl­imidazole)di-μ-propionato-penta­manganese(III)manganese(II)–di­methyl­formamide–methanol (1/0.24/1.36)

**DOI:** 10.1107/S1600536813021314

**Published:** 2013-08-10

**Authors:** Jacob C. Lutter, Jeff W. Kampf, Matthias Zeller, Curtis M. Zaleski

**Affiliations:** a Department of Chemistry, Shippensburg University, 1871 Old Main Dr., Shippensburg, PA 17257, USA; b Department of Chemistry, University of Michigan, 930 N. University Ave, Ann Arbor, MI 48109, USA; c Department of Chemistry, Youngstown State University, 1 University Plaza, Youngstown, OH 44555, USA

## Abstract

The title compound [Mn_6_(C_7_H_4_NO_3_)_5_(C_3_H_5_O_2_)_2_(C_4_H_6_N_2_)_4.17_(C_3_H_7_NO)_1.83_]·0.24C_3_H_7_NO·1.36CH_3_OH or Mn(II)(C_3_H_5_O_2_)_2_[15-MC_Mn(III)N(shi)_-5](Me—Im)_4.17_(DMF)_1.83_·0.24DMF·1.36MeOH (where MC is metallacrown, shi^3−^ is salicyl­hydroximate, Me—Im is 1-methyl­imidazole, DMF is *N*,*N*-di­methyl­formamide, and MeOH is methanol), contains an Mn^II^ ion in the central cavity and five Mn^III^ ions in the MC ring. The central Mn^II^ ion is seven coordinate and has a geometry best described as distorted face-capped trigonal prismatic with Φ angles of 6.13, 10.36, and 11.73° and an estimated average *s/h* ratio of 1.03±0.11. Four of the ring Mn^III^ ions are six coordinate with distorted octa­hedral geometries. Two of the Mn^III^ ions have Λ absolute stereoconfiguration, while the other two Mn^III^ ions have a planar configuration. The fifth Mn^III^ ion is five coordinate and has a distorted square pyramidal geometry with *τ* = 0.20. Three of the Mn^III^ ions bind one 1-methyl­imidazole ligand. Two of the ring Mn^III^ ions have a 1-methyl­imidazole and a DMF disordered over a coordination site. For one of the ring Mn^III^ ions, the occupancy ratio of the ligands refines to 0.51 (1):0.49 (1) in favor of the DMF. For the other ring Mn^III^ ion, the occupancy ratio of the ligands refines to 0.68 (1):0.32 (1) in favor of the 1-methyl­imidazole. Two propionate anions serve to bridge the central Mn^II^ ion between two different Mn^III^ ions. The methyl groups of the bridging propionate anions are disordered over two positions. The methyl group disorder also induces disorder in the H atoms of the adjacent methyl­ene C atom to the same degree. For one of the propionate anions, the occupancy ratio refines to 0.752 (8):0.248 (8) and for the second, the occupancy ratio refines to 0.604 (6):0.396 (6). In addition, the disorder of the methyl group of the latter propionate anion is correlated with a partially occupied [0.604 (6)] methanol mol­ecule. Furthermore, a methanol mol­ecule and a DMF mol­ecule are positionally disordered in the lattice. The occupancy refines to 0.757 (7):0.243 (7) in favor of the methanol mol­ecule. Correlated to the occupancy of the methanol and DMF mol­ecules is a disordered benzene ring of one salicyl­hydroximate ligand. The benzene ring is disordered over two positions with an occupancy ratio of 0.757 (7):0.243 (7). Lastly, the two lattice methanol mol­ecules are hydrogen bonded to the 15-MC-5 mol­ecule. For the partially occupied methanol mol­ecule associated with the disordered propionate anion, the hydroxyl group of the methanol is hydrogen bonded to a carboxyl­ate O atom of the propionate anion. For the partially occupied methanol mol­ecule associated with the partially occupied lattice DMF mol­ecule, the hydroxyl group of the methanol is hydrogen bonded to the phenolate O atom of a salicyl­hydroximate ligand and to the carbonyl O atom of a coordinated DMF mol­ecule.

## Related literature

For related Mn(II)[15-MC_Mn(III)N(shi)_-5)] structures and synthetic procedures, see: Kessissoglou *et al.* (1994 ▶); Dendrinou-Samara *et al.* (2001 ▶, 2002 ▶, 2005 ▶); Emerich *et al.* (2010 ▶); Tigyer *et al.* (2011 ▶, 2012 ▶, 2013 ▶). For explanations of how to calculate the *s*/*h* ratio, bond-valence-sum values and the τ parameter, see: Stiefel & Brown (1972 ▶), Liu & Thorp (1993 ▶) and Addison *et al.* (1984 ▶), respectively.
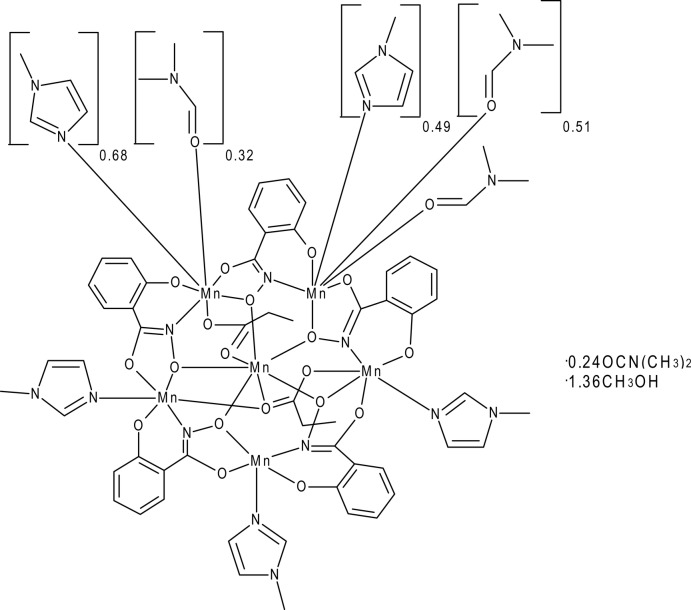


## Experimental

### 

#### Crystal data

[Mn_6_(C_7_H_4_NO_3_)_5_(C_3_H_5_O_2_)_2_(C_4_H_6_N_2_)_4.17_(C_3_H_7_NO)_1.83_]·0.24C_3_H_7_NO·1.36CH_4_O
*M* *_r_* = 1763.91
Triclinic, 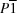

*a* = 12.6138 (2) Å
*b* = 14.8745 (3) Å
*c* = 20.7862 (15) Å
α = 97.909 (7)°
β = 105.209 (7)°
γ = 99.034 (7)°
*V* = 3650.7 (3) Å^3^
*Z* = 2
Cu *K*α radiation
μ = 8.93 mm^−1^
*T* = 85 K
0.07 × 0.02 × 0.02 mm

#### Data collection

Rigaku Saturn 944+ CCD diffractometer
Absorption correction: multi-scan (*REQAB*; Jacobson, 1998 ▶) *T*_min_ = 0.179, *T*_max_ = 0.233
106589 measured reflections
13145 independent reflections
10454 reflections with *I* > 2σ(*I*)
*R*_int_ = 0.106

#### Refinement

*R*[*F* ^2^ > 2σ(*F* ^2^)] = 0.059
*wR*(*F* ^2^) = 0.164
*S* = 1.09
13145 reflections
1153 parameters
225 restraints
H-atom parameters constrained
Δρ_max_ = 1.04 e Å^−3^
Δρ_min_ = −0.58 e Å^−3^

### 

Data collection: *CrystalClear-SM Expert* (Rigaku, 2011 ▶); cell refinement: *CrystalClear-SM Expert*; data reduction: *CrystalClear-SM Expert*; program(s) used to solve structure: *SHELXS97* (Sheldrick, 2008 ▶); program(s) used to refine structure: *SHELXL2013* (Sheldrick, 2008 ▶) and *SHELXLE* (Hübschle *et al.*, 2011 ▶); molecular graphics: *Mercury* (Macrae *et al.*, 2006 ▶); software used to prepare material for publication: *publCIF* (Westrip, 2010 ▶).

## Supplementary Material

Crystal structure: contains datablock(s) I, New_Global_Publ_Block. DOI: 10.1107/S1600536813021314/jj2172sup1.cif

Structure factors: contains datablock(s) I. DOI: 10.1107/S1600536813021314/jj2172Isup2.hkl

Additional supplementary materials:  crystallographic information; 3D view; checkCIF report

## Figures and Tables

**Table 1:** Hydrogen-bond geometry (Å, °)

*D*—H⋯*A*	*D*—H	H⋯*A*	*D*⋯*A*	*D*—H⋯*A*
O21—H21⋯O17	0.84	2.12	2.948 (6)	170
O22—H22⋯O7	0.84	2.26	3.077 (7)	163
O22—H22⋯O20	0.84	2.37	2.887 (6)	120

**Fig. 1.**
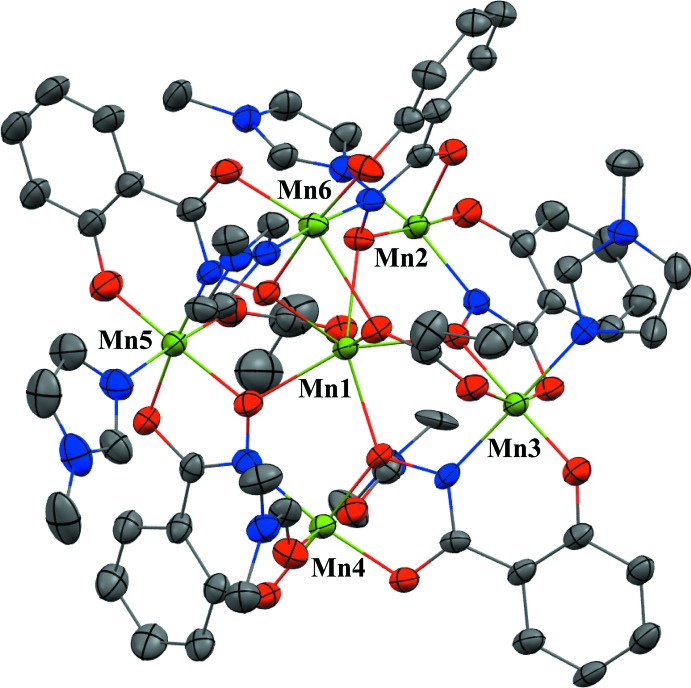
Single-crystal X-ray structure of Mn(II)(C3H5O2)2[15-MCMn(III)N(shi)-5](Me—Im)4.17(DMF)1.83·0.24DMF·1.36MeOH (1). The thermal ellipsoid plot of 1 is at a 50% probability level. For Mn4 only the DMF is shown bound to the MnIII, since the DMF possesses a higher occupancy rate compared to the coordinated 1-methylimidazole (0.51 (1):0.49 (1)). For Mn5 only the 1-methylimidazole is shown bound to the MnIII, since the 1-methylimidazole possesses a higher occupancy rate compared to the coordinated DMF (0.68 (1):0.32 (1)). The disordered benzene ring is only shown at the higher occupancy factor. Hydrogen atoms and the lattice solvent molecules have been omitted for clarity. Color scheme for all figures: green - MnII and MnIII, red - oxygen, blue - nitrogen, and gray - carbon.

**Fig. 2.**
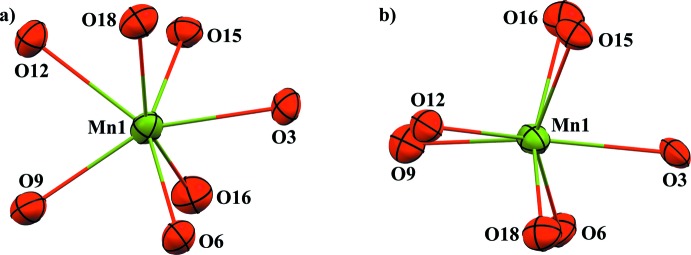
Side (a) and top (b) views of the first coordination sphere about Mn1 (2+ oxidation state) of 1. The thermal ellipsoid plots are at a 50% probability level.

**Fig. 3.**
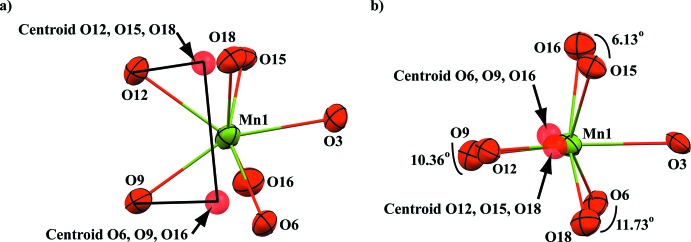
Side (a) and top (b) views of the first coordination sphere about Mn1 of 1 demonstrating how the azimuthal angle (Φ) was defined and calculated using the program Mercury (Macrae et al., 2006). The thermal ellipsoid plots are at a 50% probability level.

**Fig. 4.**
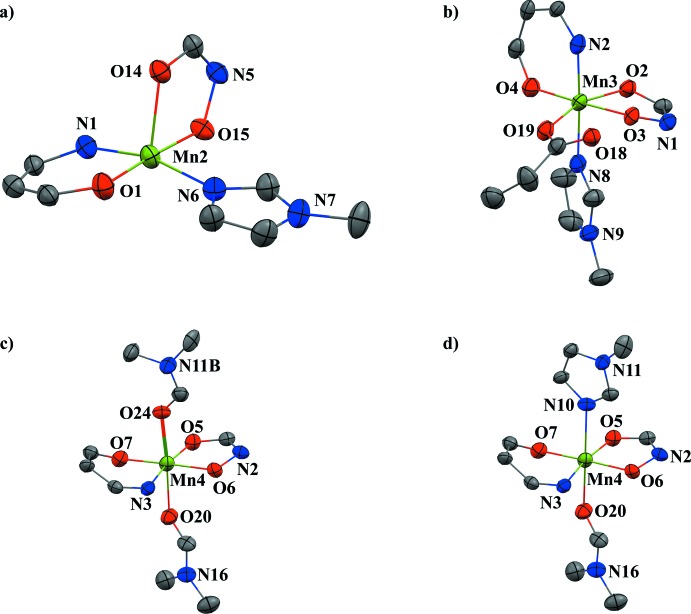
First coordination sphere about the MnIII ions Mn2, Mn3, and Mn4 of 1. a) Mn2 with distorted square pyramidal geometry b) Mn3 with Λ configuration c) Mn4 with planar configuration and DMF bound (0.51 (1) occupancy) d) Mn4 with planar configuration and 1-methylimidazole bound (0.49 (1) occupancy). The thermal ellipsoid plots are at a 50% probability level. Hydrogen atoms have been omitted for clarity.

**Fig. 5.**
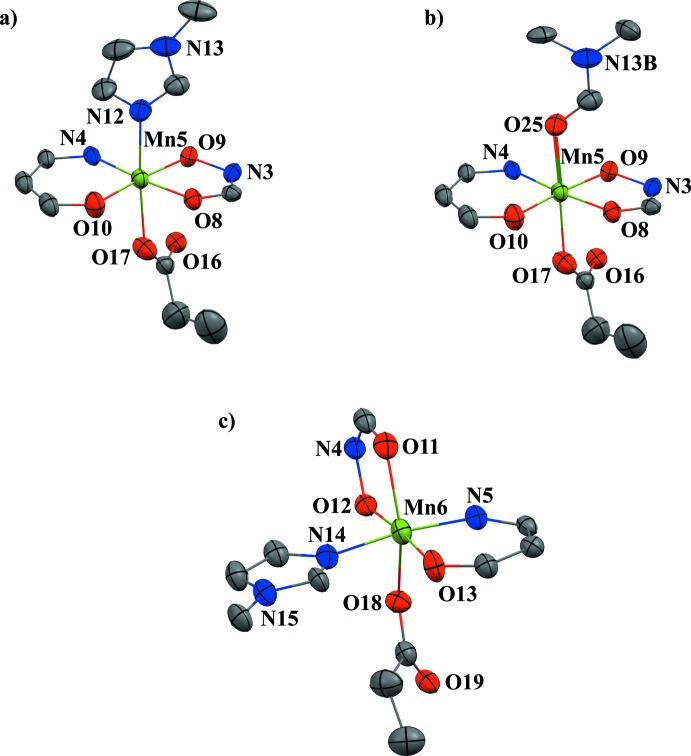
First coordination sphere about the MnIII ions Mn5 and Mn6 of 1. a) Mn5 with planar configuration and 1-methylimidazole bound (0.68 (1) occupancy) b) Mn5 with planar configuration and DMF bound (0.32 (1) occupancy) and c) Mn6 with Λ configuration. The thermal ellipsoid plots are at a 50% probability level. Hydrogen atoms have been omitted for clarity.

## supplementary crystallographic information

### 1. Comment

The first manganese 15-MC_Mn(III)N(shi)_-5 complex reported by Kessissoglou, Kampf, and Pecoraro contained six pyridine molecules bound to the ring Mn^III^ ions and two acetate anions that bridged the central Mn^II^ ion to two ring Mn^III^ ions (Kessissoglou *et al.*, 1994). Both the pyridine molecules and acetate anions serve as potential places where substitution on the 15-MC_Mn(III)N(shi)_-5 framework may occur. Initially the peripheral pyridine molecules were conserved and the bridging carboxylate anions were varied. X-ray crystal structures with 2,4-dichlorophenoxyacetate (Dendrinou-Samara *et al.*, 2001), 2,4,5-trichlorophenoxyacetate (Dendrinou-Samara *et al.*, 2002), and formate (Dendrinou-Samara *et al.*, 2005) have been reported. However, recently we have shown that the pyridine molecules can be substituted with imidazole and its derivatives while acetate anions are used as the bridging anions (Emerich *et al.*, 2010; Tigyer *et al.* 2011, 2013) or both the pyridine molecules and acetate anions can be substituted with imidazole molecules and bridging formate anions (Tigyer *et al.* 2012). Herein we report the synthesis, IR data, and single-crystal X-ray structure of the title compound [Mn_6_(C_7_H_4_NO_3_)_5_(C_3_H_5_O_2_)_2_(C_4_H_6_N_2_)_4.17_(C_3_H_7_NO)_1.83_]·0.24C_3_H_7_NO·1.36CH_3_OH, 1, abbreviated as Mn(II)(C_3_H_5_O_2_)_2_[15-MC_Mn(III)N(shi)_-5](Me—Im)_4.17_(DMF)_1.83_·0.24DMF·1.36MeOH (where MC is metallacrown, shi^3-^ is salicylhydroximate, Me—Im is 1-methylimidazole, DMF is *N*,*N*-dimethylformamide, and MeOH is methanol). In 1 propionate serves as the bridging carboxylate anion and 1-methylimidazole is bound to the ring Mn^III^ ions instead of pyridine.

The overall metallacrown structure of 1 is nonplanar, which is typical of manganese-based 15-MC-5 complexes (Fig. 1). The MC framework is built from five shi^3-^ ligands and five Mn^III^ ions which form a –[Mn^III^—N—O]- repeat unit. Located in the central cavity of the MC is a Mn^II^ ion. This Mn^II^ is bound to five oxime O atoms of the shi^3-^ and is also connected to the MC framework *via* two bridging propionate anions. Charge neutrality is maintained for the MC by the five Mn^III^ cations, one Mn^II^ cation, and the five shi^3-^ and two propionate anions.

The central manganese ion (Mn1) is seven coordinate with a geometry best described as distorted face-capped trigonal prismatic (Fig. 2). The coordination of Mn1 is completed by five oxime oxygen atoms of the shi^3-^ ligands and two carboxylate oxygen atoms of two different propionate anions. The two propionate anions bridge the central Mn1 to Mn3 and Mn5 of the MC ring. The geometry assignment is supported by both the azimuthal angle, Φ, and the calculated *s/h* ratio (Stiefel & Brown, 1972). These parameters are used to distinguish between octahedral and trigonal prismatic geometry. In an ideal octahedron, the angle between the atoms on opposite triganular faces is Φ = 60°, and the *s/h* ratio is 1.22. In an ideal trigonal prism, the azimuthal angle is 0°, and the *s/h* ratio is 1.00. To calculate these parameters the centroids of opposite triangular faces made by the donor oxygen atoms (O6, O9, and O16; O12, O15, and O18) were defined using the program *Mercury* (Fig. 3; Macrae *et al.*, 2006). The azimuthal angles were measured between atoms on opposite faces through the centroids. To calculate the *s/h* ratio, the distance between the centroids was defined as *h*, and the distances between atoms on the same triangular face were defined as *s*. For Mn1 the Φ angles are 6.13°, 10.36°, and 11.73°, and the estimated average *s/h* ratio is 1.03±0.11. Thus, both the Φ angle and *s/h* ratio support a distorted faced-capped trigonal prismatic geometry. Mn1 is assigned a 2+ oxidation state, which is supported by a Bond Valence Sum (BVS) value of 1.93 (Liu & Thorp, 1993) and an average Mn—O bond distance of 2.24 Å.

The five ring Mn ions possess various coordination numbers and configuration modes (Fig. 4 and 5). Mn2 has a coordination number of five and possesses a distorted square pyramidal geometry (Fig. 4a). To evaluate the geometry about Mn2 the *τ* parameter was calculated (Addison *et al.*, 1984). For an ideal square pyramidal geometry *τ* = 0, while for an ideal trigonal bipyramidal geometry *τ* = 1. For Mn2 the *τ* parameter is 0.20. Mn3 - Mn6 are six-coordinate with distorted octahedral geometry, but the configuration of the coordination about each Mn is different. Mn3 has a propeller configuration of two chelate rings of different shi^3-^ ligands with Λ absolute stereochemistry (Fig. 4b). In addition, Mn3 binds one 1-methylimidazole ligand. Mn4 has a planar configuration, where two chelate rings of different shi^3-^ ligands are located *trans* to each other (Fig. 4c and 4d). Along the axial axis Mn4 also binds a DMF molecule, and located in a *trans* position is either a 1-methylimidazole or a DMF. For the ligands bound to Mn4, the occupancy ratio refines to 0.51 (1) to 0.49 (1) in favor of the DMF molecule. Mn5 also has a planar configuration of two *trans* chelate rings of different shi^3-^ ligands (Fig. 5a and 5b). Along the axial axis is an carboxylate oxygen atom of a propionate ligand and located in a *trans* position is either a 1-methylimidazole or a DMF. For the ligands bound Mn5, the occupancy ratio refines to 0.68 (1) to 0.32 (1) in favor of the 1-methylimidazole molecule. Mn6 has a propeller configuration of two chelate rings of different shi^3-^ ligands with Λ absolute stereochemistry (Fig. 5c). In addition, Mn6 binds one 1-methylimidazole ligand. Mn2, Mn3, Mn4, Mn5, and Mn6 are assigned a 3+ oxidation state based on BVS values of 2.97, 3.12, 3.11, 3.19, 3.09, respectively, and average bond Mn—N/O distances of 1.98, 2.03, 2.02, 2.03, 2.06 Å, respectively. In addition, the oxidation state assignment is further supported by the presence of a Jahn-Teller axis for Mn3 – Mn6, which is typical of high spin *d*^4^ cations.

Lastly, several more instances of disorder exist in the structure. The methyl groups of the bridging propionate anions are disordered over two positions. The methyl group disorder also induces disorder in the hydrogen atoms of the adjacent methylene carbon atom to the same degree. For the propionate anion that bridges Mn1 to Mn3, the occupancy ratio refines to 0.752 (8) to 0.248 (8). For the propionate anion that bridges Mn1 to Mn5, the occupancy ratio refines to 0.604 (6) to 0.396 (6). In addition, the disorder of the methyl group of the latter propionate anion is correlated with a partially occupied methanol molecule. The occupancy of the methanol molecule is 0.604 (6). Furthermore, a methanol molecule and a DMF molecule are positionally disordered in the lattice with an occupancy ratio of 0.757 (7) to 0.243 (7) in favor of the methanol molecule. Correlated to the occupancy of the methanol and DMF molecules is a disordered benzene ring (C15 to C20 and C15B to C20B) of one salicylhydroximate ligand. The benzene ring is disordered over two positions with an occupancy ratio of 0.757 (7) to 0.243 (7).

### 2. Experimental

Manganese(II) chloride tetrahydrate (99%), salicylhydroxamic acid (H_3_shi, 99%), sodium propionate (99%), and 1-methylimidazole (99%) were purchased from Alfa Aesar. Sodium methoxide was purchased from Matheson Coleman and Bell. Methanol (HPLC grade) was purchased from Pharmco-AAPer. *N*,*N*-dimethylformamide (Certified ACS grade) was purchased from BDH chemicals. All reagents were used as received and without further purification.

Manganese(II) chloride tetrahydrate (0.75 mmol) was dissolved in 12.5 ml of methanol resulting in a light pink solution. Sodium methoxide (1.875 mmol) and H_3_shi (0.625 mmol) were then added to the manganese(II) chloride solution. Initially the solution turned a yellow color, but after stirring for 1 h the solution became dark brown. After 1 h of stirring, neat 1-methylimidazole (2.5 mmol) and a mixture of sodium propionate (0.75 mmol) in 12.5 ml of DMF were added to the dark brown solution. No color change was observed. After 5 minutes of stirring, the solution was filtered and the filtrate was left for slow evaporation of the solvent at room temperature. Dark brown-black crystals suitable for X-ray diffraction analysis were collected after 8 days. The percent yield was 5.0% based on manganese(II) chloride tetrahydrate. Elemental analysis for the dried material C_65.264_H_74.971_Mn_6_N_15.417_O_22.430_ [FW = 1763.91 g/mol] found % (calculated); C 44.18 (44.44); H 4.00 (4.28); N 12.46 (12.24).

### 3. Refinement

For Mn4 and Mn5, a 1-methylimidazole molecule and a *N*,*N*-dimethylformamide (DMF) molecule are disordered over a coordination site. Overlapping atoms were constrained to have identical anisotropic displacement parameters (ADPs). The 1-methylimidazole molecules were restrained to have geometries similar to that of another non-disordered 1-methylimidazole. The DMF molecules were also restrained to have a geometry similar to that of another non-disordered DMF molecule. Carbon and nitrogen atoms of the 1-methylimidazole molecules and carbon, nitrogen, and oxygen atoms of the DMF molecules were restrained to have similar *U_ij_* components of the ADPs (e.s.d. = 0.01 Å^2^; SIMU restraint in Shexltl). For the ligands bound to Mn4, the occupancy ratio refined to 0.506 (12) to 0.494 (12) in favor of the DMF molecule. For the ligands bound Mn5, the occupancy ratio refined to 0.680 (12) to 0.320 (12) in favor of the 1-methylimidazole molecule.

The methyl groups of the bridging propionate ligands are disordered over two positions. The methyl group disorder also induces disorder in the hydrogen atoms of the adjacent methylene carbon atom to the same degree. The ADPs for each pair of methyl groups were constrained to be identical. For the propionate that bridges Mn1 to Mn3 the occupancy ratio refined to 0.752 (8) to 0.248 (8). For the propionate that bridges Mn1 to Mn5 the occupancy ratio refined to 0.604 (6) to 0.396 (6). In addition, the disorder of the methyl group of the latter propionate anion is correlated with a partially occupied methanol molecule. The occupancy of the methanol molecule is also 0.604 (6).

A methanol molecule and a DMF molecule are positional disordered in the lattice. Carbon, nitrogen, and oxygen atoms of the DMF molecule were restrained to have similar *U_ij_* components of the ADPs (e.s.d. = 0.01 Å^2^; SIMU restraint in Shexltl). The occupancy refined to 0.757 (7) to 0.243 (7) in favor of the methanol molecule.

Correlated to the occupancy of the methanol and DMF molecules is a disordered benzene ring (C15 to C20 and C15B to C20B) of one salicylhydroximate ligand. The benzene ring is disordered over two positions with an occupancy ratio of 0.757 (7) to 0.243 (7). Equivalent atoms of the benzene ring have nearly the same atom positions leading to highly correlated thermal parameters. To avoid correlation, the ADPs of every pair of overlapping atoms were constrained to be identical. For the disordered benzene ring carbon atoms that connect to the non-disordered portion of the salicylhydroximate ligand, carbon-carbon (C20—C21 and C20B—C21) and carbon-oxygen (C15—O7 and C15B—O7) bond distances were restrained to be similar (e.s.d. = 0.02 Å). To maintain the planarity of each disordered benzene ring, the chiral volumes of the carbon atoms (C15, C15B, C20, and C20B) that connect to the non-disordered portion of the salicylhydroximate ligand were restrained to zero (e.s.d. = 0.1 Å^3^).

All hydrogen atoms were placed in calculated positions and refined as riding on their carrier atoms with O—H distances of 0.84 Å for methanol oxygen atoms and C—H distances of 0.95 Å for *sp*^2^ carbon atoms, 0.99 Å for methylene carbon atoms, and 0.98 Å for methyl carbon atoms. The *U*_iso_ values for hydrogen atoms were set to a multiple of the value of the carrying carbon atom (1.2 times for *sp*^2^ hybridized carbon atoms and methylene carbon atoms or 1.5 times for methyl carbon atoms and methanol oxygen atoms).

One low angle reflection (0 1 0) was affected by the beam stop and omitted from the refinement.

### Crystal data

**Table:**

[Mn_6_(C_7_H_4_NO_3_)_5_(C_3_H_5_O_2_)_2_(C_4_H_6_N_2_)_4.17_(C_3_H_7_NO)_1.83_]·0.24C_3_H_7_NO·1.36CH_4_O	*Z* = 2
*M**_r_* = 1763.91	*F*(000) = 1807.8
Triclinic, *P*1	*D*_x_ = 1.604 Mg m^−^^3^
Hall symbol: -P 1	Cu *K*α radiation, λ = 1.54178 Å
*a* = 12.6138 (2) Å	Cell parameters from 45007 reflections
*b* = 14.8745 (3) Å	θ = 2.2–68.3°
*c* = 20.7862 (15) Å	µ = 8.93 mm^−^^1^
α = 97.909 (7)°	*T* = 85 K
β = 105.209 (7)°	Needle, brown
γ = 99.034 (7)°	0.07 × 0.02 × 0.02 mm
*V* = 3650.7 (3) Å^3^	

### Data collection

**Table:**

Rigaku Saturn 944+ CCD diffractometer	10454 reflections with *I* > 2σ(*I*)
Radiation source: micro-focus rotating anode	*R*_int_ = 0.106
ω scans	θ_max_ = 68.3°, θ_min_ = 2.2°
Absorption correction: multi-scan (*REQAB*; Jacobson, 1998)	*h* = −15→15
*T*_min_ = 0.179, *T*_max_ = 0.233	*k* = −17→17
106589 measured reflections	*l* = −25→25
13145 independent reflections	

### Refinement

**Table:**

Refinement on *F*^2^	Secondary atom site location: difference Fourier map
Least-squares matrix: full	Hydrogen site location: inferred from neighbouring sites
*R*[*F*^2^ > 2σ(*F*^2^)] = 0.059	H-atom parameters constrained
*wR*(*F*^2^) = 0.164	*w* = 1/[σ^2^(*F*_o_^2^) + (0.1023*P*)^2^] where *P* = (*F*_o_^2^ + 2*F*_c_^2^)/3
*S* = 1.09	(Δ/σ)_max_ = 0.003
13145 reflections	Δρ_max_ = 1.04 e Å^−^^3^
1153 parameters	Δρ_min_ = −0.58 e Å^−^^3^
225 restraints	Extinction correction: *SHELXL*, Fc^*^=kFc[1+0.001xFc^2^λ^3^/sin(2θ)]^-1/4^
Primary atom site location: structure-invariant direct methods	Extinction coefficient: 0.00166 (14)

### Special details

**Table:**

Experimental. FT–IR bands (KBr pellet, cm^-1^): 1654, 1598, 1571, 1541, 1499, 1467, 1436, 1420, 1388, 1321, 1259, 1244, 1146, 1101, 1032, 1024, 953, 926, 866, 836, 754, 679, 670, 648, 618, 596, 576, 538, 467
Geometry. All e.s.d.'s (except the e.s.d. in the dihedral angle between two l.s. planes) are estimated using the full covariance matrix. The cell e.s.d.'s are taken into account individually in the estimation of e.s.d.'s in distances, angles and torsion angles; correlations between e.s.d.'s in cell parameters are only used when they are defined by crystal symmetry. An approximate (isotropic) treatment of cell e.s.d.'s is used for estimating e.s.d.'s involving l.s. planes.

### Fractional atomic coordinates and isotropic or equivalent isotropic displacement parameters (Å^2^)

**Table:**

	*x*	*y*	*z*	*U*_iso_*/*U*_eq_	Occ. (<1)
Mn1	0.07000 (5)	0.73648 (4)	0.24929 (3)	0.02589 (16)	
Mn2	0.11769 (5)	0.79206 (4)	0.41954 (3)	0.02689 (16)	
Mn3	0.13864 (5)	0.52190 (4)	0.28707 (3)	0.02769 (17)	
Mn4	0.27830 (5)	0.71726 (4)	0.15708 (3)	0.03072 (17)	
Mn5	0.06535 (5)	0.94096 (4)	0.18825 (3)	0.03079 (17)	
Mn6	−0.20256 (5)	0.73342 (4)	0.23549 (3)	0.03058 (17)	
O1	0.2247 (2)	0.80548 (18)	0.50183 (15)	0.0350 (6)	
O2	0.2761 (2)	0.57455 (17)	0.37874 (14)	0.0298 (6)	
O3	0.1073 (2)	0.63805 (17)	0.31920 (13)	0.0272 (6)	
O4	0.1788 (2)	0.41077 (18)	0.26075 (15)	0.0359 (7)	
O5	0.3234 (2)	0.59673 (18)	0.15415 (15)	0.0343 (6)	
O6	0.1851 (2)	0.66244 (16)	0.20613 (14)	0.0296 (6)	
O7	0.3659 (2)	0.77049 (18)	0.10658 (15)	0.0358 (7)	
O8	0.2015 (2)	0.97961 (17)	0.16313 (15)	0.0341 (6)	
O9	0.1062 (2)	0.82092 (18)	0.17407 (15)	0.0328 (6)	
O10	0.0368 (3)	1.0586 (2)	0.20814 (18)	0.0486 (8)	
O11	−0.2213 (2)	0.8757 (2)	0.24916 (15)	0.0374 (7)	
O12	−0.0858 (2)	0.79252 (17)	0.20389 (14)	0.0301 (6)	
O13	−0.3130 (2)	0.6622 (2)	0.26136 (15)	0.0398 (7)	
O14	−0.0355 (2)	0.73088 (17)	0.43793 (14)	0.0294 (6)	
O15	0.0091 (2)	0.78722 (17)	0.33613 (14)	0.0288 (6)	
O16	0.2080 (2)	0.83738 (19)	0.32964 (16)	0.0383 (7)	
O17	0.1796 (3)	0.9699 (2)	0.29502 (17)	0.0479 (8)	
O18	−0.0884 (2)	0.62742 (17)	0.20646 (15)	0.0334 (6)	
O19	−0.0280 (2)	0.49444 (18)	0.21104 (15)	0.0343 (6)	
N1	0.1634 (3)	0.6774 (2)	0.38746 (17)	0.0286 (7)	
N2	0.2095 (3)	0.5772 (2)	0.22215 (18)	0.0299 (7)	
N3	0.2138 (3)	0.8296 (2)	0.16412 (18)	0.0321 (7)	
N4	−0.0672 (3)	0.8893 (2)	0.21208 (17)	0.0309 (7)	
N5	−0.1029 (2)	0.7460 (2)	0.32834 (17)	0.0293 (7)	
N6	0.1097 (3)	0.9275 (2)	0.44749 (17)	0.0308 (7)	
N7	0.0641 (3)	1.0626 (2)	0.44134 (18)	0.0353 (8)	
N8	0.0546 (3)	0.4610 (2)	0.34773 (18)	0.0304 (7)	
N9	−0.0657 (3)	0.4385 (2)	0.40582 (19)	0.0380 (8)	
C1	0.2989 (3)	0.7533 (3)	0.5255 (2)	0.0313 (9)	
C2	0.3615 (4)	0.7793 (3)	0.5931 (2)	0.0398 (10)	
H2	0.3515	0.8325	0.6200	0.048*	
C3	0.4384 (4)	0.7286 (3)	0.6218 (3)	0.0483 (11)	
H3	0.4804	0.7470	0.6683	0.058*	
C4	0.4547 (4)	0.6507 (3)	0.5829 (3)	0.0456 (11)	
H4	0.5073	0.6157	0.6026	0.055*	
C5	0.3936 (3)	0.6252 (3)	0.5158 (2)	0.0356 (9)	
H5	0.4051	0.5723	0.4893	0.043*	
C6	0.3149 (3)	0.6748 (3)	0.4852 (2)	0.0290 (8)	
C7	0.2508 (3)	0.6403 (2)	0.4139 (2)	0.0277 (8)	
C8	0.2608 (3)	0.3953 (3)	0.2335 (2)	0.0322 (9)	
C9	0.2950 (4)	0.3100 (3)	0.2378 (2)	0.0361 (9)	
H9	0.2622	0.2678	0.2609	0.043*	
C10	0.3763 (4)	0.2878 (3)	0.2084 (2)	0.0431 (11)	
H10	0.3980	0.2298	0.2110	0.052*	
C11	0.4268 (4)	0.3487 (3)	0.1753 (2)	0.0400 (10)	
H11	0.4832	0.3328	0.1558	0.048*	
C12	0.3945 (3)	0.4325 (3)	0.1708 (2)	0.0364 (9)	
H12	0.4285	0.4739	0.1476	0.044*	
C13	0.3118 (3)	0.4580 (3)	0.2001 (2)	0.0299 (8)	
C14	0.2813 (3)	0.5478 (3)	0.1920 (2)	0.0287 (8)	
C21	0.2552 (3)	0.9130 (2)	0.1573 (2)	0.0304 (8)	
C22	−0.0456 (4)	1.0874 (3)	0.2287 (2)	0.0417 (10)	
C23	−0.0429 (5)	1.1827 (3)	0.2382 (3)	0.0498 (12)	
H23	0.0166	1.2233	0.2304	0.060*	
C24	−0.1265 (5)	1.2187 (3)	0.2591 (3)	0.0554 (14)	
H24	−0.1226	1.2837	0.2666	0.066*	
C25	−0.2155 (5)	1.1598 (4)	0.2689 (3)	0.0626 (16)	
H25	−0.2738	1.1841	0.2816	0.075*	
C26	−0.2185 (4)	1.0656 (3)	0.2599 (3)	0.0496 (12)	
H26	−0.2790	1.0257	0.2673	0.060*	
C27	−0.1346 (4)	1.0276 (3)	0.2402 (2)	0.0385 (10)	
C28	−0.1435 (3)	0.9263 (3)	0.2338 (2)	0.0334 (9)	
C29	−0.3222 (3)	0.6498 (3)	0.3222 (2)	0.0333 (9)	
C30	−0.4272 (3)	0.6082 (3)	0.3263 (2)	0.0362 (9)	
H30	−0.4882	0.5893	0.2860	0.043*	
C31	−0.4446 (3)	0.5938 (3)	0.3875 (2)	0.0393 (10)	
H31	−0.5169	0.5658	0.3888	0.047*	
C32	−0.3566 (3)	0.6203 (3)	0.4467 (2)	0.0385 (10)	
H32	−0.3680	0.6108	0.4889	0.046*	
C33	−0.2520 (3)	0.6606 (3)	0.4438 (2)	0.0328 (9)	
H33	−0.1917	0.6778	0.4845	0.039*	
C34	−0.2320 (3)	0.6769 (2)	0.3831 (2)	0.0299 (9)	
C35	−0.1174 (3)	0.7194 (2)	0.3844 (2)	0.0273 (8)	
C36	0.2347 (3)	0.9241 (3)	0.3333 (2)	0.0313 (9)	
C37	0.3362 (5)	0.9792 (5)	0.3857 (4)	0.0814 (19)	
H37A	0.3668	0.9391	0.4172	0.098*	0.604 (6)
H37B	0.3147	1.0301	0.4123	0.098*	0.604 (6)
H37C	0.4044	0.9650	0.3748	0.098*	0.396 (6)
H37D	0.3373	0.9636	0.4306	0.098*	0.396 (6)
C38	0.4254 (11)	1.0193 (7)	0.3574 (7)	0.094 (3)	0.604 (6)
H38A	0.4316	0.9732	0.3208	0.141*	0.604 (6)
H38B	0.4971	1.0371	0.3932	0.141*	0.604 (6)
H38C	0.4063	1.0741	0.3394	0.141*	0.604 (6)
C38B	0.3341 (17)	1.0933 (11)	0.3875 (11)	0.094 (3)	0.396 (6)
H38D	0.3282	1.1073	0.3421	0.141*	0.396 (6)
H38E	0.4034	1.1307	0.4197	0.141*	0.396 (6)
H38F	0.2695	1.1077	0.4017	0.141*	0.396 (6)
C39	−0.1033 (3)	0.5398 (3)	0.1960 (2)	0.0336 (9)	
C40	−0.2234 (4)	0.4873 (3)	0.1624 (3)	0.0538 (13)	
H40A	−0.2730	0.5179	0.1841	0.065*	0.752 (8)
H40B	−0.2441	0.4939	0.1142	0.065*	0.752 (8)
H40C	−0.2263	0.4394	0.1236	0.065*	0.248 (8)
H40D	−0.2716	0.5305	0.1454	0.065*	0.248 (8)
C41	−0.2477 (6)	0.3871 (4)	0.1648 (4)	0.0576 (19)	0.752 (8)
H41A	−0.3271	0.3610	0.1414	0.086*	0.752 (8)
H41B	−0.2310	0.3791	0.2122	0.086*	0.752 (8)
H41C	−0.2011	0.3550	0.1424	0.086*	0.752 (8)
C41B	−0.2672 (17)	0.4393 (14)	0.2188 (13)	0.0576 (19)	0.248 (8)
H41D	−0.3412	0.3992	0.1971	0.086*	0.248 (8)
H41E	−0.2725	0.4875	0.2544	0.086*	0.248 (8)
H41F	−0.2142	0.4021	0.2390	0.086*	0.248 (8)
C42	0.0481 (4)	0.9756 (3)	0.4090 (2)	0.0360 (9)	
H42	−0.0006	0.9514	0.3646	0.043*	
C43	0.1677 (4)	0.9875 (3)	0.5072 (2)	0.0371 (10)	
H43	0.2190	0.9727	0.5447	0.044*	
C44	0.1393 (4)	1.0716 (3)	0.5037 (2)	0.0388 (10)	
H44	0.1664	1.1260	0.5379	0.047*	
C45	0.0103 (5)	1.1352 (3)	0.4142 (3)	0.0507 (12)	
H45A	0.0597	1.1719	0.3939	0.076*	
H45B	−0.0041	1.1756	0.4510	0.076*	
H45C	−0.0609	1.1068	0.3796	0.076*	
C46	−0.0308 (4)	0.4887 (3)	0.3642 (2)	0.0407 (11)	
H46	−0.0631	0.5381	0.3485	0.049*	
C47	0.0756 (4)	0.3903 (3)	0.3818 (3)	0.0485 (12)	
H47	0.1330	0.3564	0.3802	0.058*	
C48	0.0019 (4)	0.3762 (3)	0.4182 (3)	0.0486 (12)	
H48	−0.0019	0.3317	0.4466	0.058*	
C49	−0.1612 (4)	0.4465 (3)	0.4325 (3)	0.0585 (15)	
H49A	−0.1903	0.5011	0.4204	0.088*	
H49B	−0.1367	0.4528	0.4820	0.088*	
H49C	−0.2202	0.3908	0.4128	0.088*	
N12	−0.036 (3)	0.914 (3)	0.0791 (10)	0.0446 (19)	0.680 (12)
C54	−0.0112 (16)	0.8711 (18)	0.0277 (9)	0.0469 (18)	0.680 (12)
H54	0.0541	0.8455	0.0316	0.056*	0.680 (12)
N13	−0.0886 (19)	0.867 (3)	−0.0311 (9)	0.0543 (14)	0.680 (12)
C55	−0.1294 (6)	0.9483 (6)	0.0511 (4)	0.055 (2)	0.680 (12)
H55	−0.1642	0.9868	0.0759	0.066*	0.680 (12)
C56	−0.1641 (8)	0.9190 (7)	−0.0158 (5)	0.064 (2)	0.680 (12)
H56A	−0.2277	0.9314	−0.0469	0.077*	0.680 (12)
C57	−0.0855 (16)	0.8253 (14)	−0.0982 (8)	0.074 (5)	0.680 (12)
H57A	−0.1461	0.7706	−0.1168	0.110*	0.680 (12)
H57B	−0.0131	0.8070	−0.0946	0.110*	0.680 (12)
H57C	−0.0951	0.8705	−0.1283	0.110*	0.680 (12)
O25	−0.039 (4)	0.924 (4)	0.0807 (18)	0.0446 (19)	0.320 (12)
C54B	−0.018 (3)	0.882 (4)	0.0319 (18)	0.0469 (18)	0.320 (12)
H54B	0.0507	0.8617	0.0395	0.056*	0.320 (12)
N13B	−0.086 (4)	0.864 (7)	−0.031 (2)	0.0543 (14)	0.320 (12)
C56B	−0.2020 (14)	0.8773 (14)	−0.0439 (10)	0.054 (5)	0.320 (12)
H56B	−0.2534	0.8209	−0.0720	0.081*	0.320 (12)
H56C	−0.2106	0.9293	−0.0678	0.081*	0.320 (12)
H56D	−0.2195	0.8908	−0.0008	0.081*	0.320 (12)
C57B	−0.056 (3)	0.814 (3)	−0.0871 (16)	0.054 (7)	0.320 (12)
H57D	−0.1133	0.7577	−0.1085	0.080*	0.320 (12)
H57E	0.0169	0.7978	−0.0702	0.080*	0.320 (12)
H57F	−0.0527	0.8541	−0.1206	0.080*	0.320 (12)
N14	−0.3112 (3)	0.7020 (2)	0.13996 (18)	0.0325 (7)	
C58	−0.4179 (3)	0.6591 (3)	0.1215 (2)	0.0320 (9)	
H58	−0.4572	0.6392	0.1520	0.038*	
N15	−0.4631 (3)	0.6474 (2)	0.05413 (18)	0.0357 (8)	
C59	−0.2875 (3)	0.7203 (3)	0.0815 (2)	0.0377 (10)	
H59	−0.2173	0.7515	0.0791	0.045*	
C60	−0.3807 (4)	0.6866 (3)	0.0275 (2)	0.0414 (10)	
H60	−0.3878	0.6894	−0.0188	0.050*	
C61	−0.5791 (3)	0.6041 (3)	0.0159 (2)	0.0414 (10)	
H61A	−0.6161	0.6498	−0.0060	0.062*	
H61B	−0.6191	0.5814	0.0469	0.062*	
H61C	−0.5798	0.5521	−0.0188	0.062*	
O20	0.1453 (2)	0.6549 (2)	0.05646 (15)	0.0403 (7)	
C62	0.0426 (4)	0.6409 (3)	0.0485 (2)	0.0366 (10)	
H62	0.0173	0.6545	0.0873	0.044*	
N16	−0.0334 (3)	0.6084 (2)	−0.01069 (19)	0.0385 (8)	
C63	−0.0017 (4)	0.5873 (3)	−0.0728 (3)	0.0497 (12)	
H63A	−0.0269	0.5209	−0.0917	0.075*	
H63B	0.0801	0.6042	−0.0626	0.075*	
H63C	−0.0369	0.6228	−0.1059	0.075*	
C64	−0.1529 (4)	0.5894 (4)	−0.0171 (3)	0.0538 (13)	
H64A	−0.1903	0.6284	−0.0463	0.081*	
H64B	−0.1641	0.6029	0.0278	0.081*	
H64C	−0.1849	0.5239	−0.0373	0.081*	
C15	0.4119 (8)	0.8609 (4)	0.1168 (5)	0.0337 (13)	0.757 (7)
C16	0.5128 (7)	0.8860 (5)	0.1005 (4)	0.037 (2)	0.757 (7)
H16	0.5470	0.8387	0.0841	0.044*	0.757 (7)
C17	0.5629 (6)	0.9772 (5)	0.1078 (4)	0.052 (3)	0.757 (7)
H17	0.6317	0.9924	0.0973	0.062*	0.757 (7)
C18	0.5129 (6)	1.0478 (5)	0.1307 (4)	0.051 (2)	0.757 (7)
H18	0.5464	1.1110	0.1348	0.061*	0.757 (7)
C19	0.4149 (7)	1.0248 (6)	0.1473 (4)	0.041 (2)	0.757 (7)
H19	0.3819	1.0731	0.1636	0.049*	0.757 (7)
C20	0.3621 (5)	0.9329 (7)	0.1411 (5)	0.0326 (13)	0.757 (7)
O22	0.2006 (5)	0.7811 (4)	−0.0291 (3)	0.0749 (18)	0.757 (7)
H22	0.2329	0.7719	0.0095	0.112*	0.757 (7)
C69	0.2756 (12)	0.8416 (10)	−0.0506 (8)	0.062 (4)	0.757 (7)
H69A	0.3010	0.9006	−0.0183	0.093*	0.757 (7)
H69B	0.2379	0.8525	−0.0956	0.093*	0.757 (7)
H69C	0.3405	0.8140	−0.0529	0.093*	0.757 (7)
C15B	0.419 (2)	0.8580 (12)	0.1257 (14)	0.0337 (13)	0.243 (7)
C16B	0.527 (3)	0.8838 (17)	0.1208 (18)	0.037 (2)	0.243 (7)
H16B	0.5640	0.8377	0.1055	0.044*	0.243 (7)
C17B	0.581 (2)	0.9745 (17)	0.1378 (16)	0.052 (3)	0.243 (7)
H17B	0.6531	0.9914	0.1315	0.062*	0.243 (7)
C18B	0.532 (2)	1.0429 (15)	0.1642 (14)	0.051 (2)	0.243 (7)
H18B	0.5716	1.1053	0.1786	0.061*	0.243 (7)
C19B	0.425 (2)	1.019 (2)	0.1694 (18)	0.041 (2)	0.243 (7)
H19B	0.3900	1.0658	0.1861	0.049*	0.243 (7)
C20B	0.3677 (14)	0.928 (2)	0.1504 (14)	0.0326 (13)	0.243 (7)
O23	0.4316 (18)	0.7602 (14)	−0.0788 (10)	0.090 (6)	0.243 (7)
C67	0.213 (4)	0.753 (3)	−0.077 (2)	0.119 (10)	0.243 (7)
H67A	0.2202	0.7202	−0.0385	0.179*	0.243 (7)
H67B	0.2282	0.7140	−0.1145	0.179*	0.243 (7)
H67C	0.1375	0.7646	−0.0917	0.179*	0.243 (7)
N17	0.297 (5)	0.843 (4)	−0.056 (3)	0.100 (8)	0.243 (7)
C66	0.272 (3)	0.926 (2)	−0.0303 (17)	0.104 (9)	0.243 (7)
H66A	0.2146	0.9430	−0.0657	0.156*	0.243 (7)
H66B	0.3401	0.9750	−0.0163	0.156*	0.243 (7)
H66C	0.2444	0.9189	0.0088	0.156*	0.243 (7)
C68	0.407 (3)	0.828 (2)	−0.0581 (17)	0.095 (7)	0.243 (7)
H68	0.4666	0.8805	−0.0405	0.114*	0.243 (7)
N10	0.4262 (18)	0.772 (3)	0.2570 (11)	0.036 (2)	0.494 (12)
C50	0.4184 (17)	0.773 (2)	0.3185 (10)	0.032 (3)	0.494 (12)
H50	0.3524	0.7464	0.3285	0.038*	0.494 (12)
N11	0.5146 (15)	0.8151 (13)	0.3657 (8)	0.034 (3)	0.494 (12)
C51	0.5333 (6)	0.8194 (6)	0.2651 (4)	0.034 (2)	0.494 (12)
H51	0.5629	0.8320	0.2290	0.041*	0.494 (12)
C52	0.5906 (9)	0.8454 (8)	0.3321 (6)	0.035 (2)	0.494 (12)
H52	0.6662	0.8773	0.3516	0.043*	0.494 (12)
C53	0.524 (2)	0.833 (2)	0.4384 (10)	0.052 (5)	0.494 (12)
H53A	0.4510	0.8089	0.4449	0.078*	0.494 (12)
H53B	0.5459	0.8995	0.4562	0.078*	0.494 (12)
H53C	0.5802	0.8013	0.4626	0.078*	0.494 (12)
O24	0.4139 (14)	0.773 (2)	0.2456 (8)	0.036 (2)	0.506 (12)
C50B	0.4263 (18)	0.778 (2)	0.3068 (10)	0.038 (3)	0.506 (12)
H50B	0.3710	0.7413	0.3209	0.046*	0.506 (12)
N11B	0.5135 (16)	0.8315 (13)	0.3535 (8)	0.041 (4)	0.506 (12)
C52B	0.6030 (11)	0.8873 (11)	0.3342 (8)	0.057 (4)	0.506 (12)
H52A	0.5856	0.8764	0.2846	0.086*	0.506 (12)
H52B	0.6746	0.8695	0.3536	0.086*	0.506 (12)
H52C	0.6086	0.9531	0.3514	0.086*	0.506 (12)
C53B	0.538 (2)	0.828 (2)	0.4263 (9)	0.047 (4)	0.506 (12)
H53D	0.4807	0.7802	0.4330	0.071*	0.506 (12)
H53E	0.5375	0.8881	0.4517	0.071*	0.506 (12)
H53F	0.6119	0.8122	0.4424	0.071*	0.506 (12)
O21	0.2053 (6)	1.1718 (4)	0.3353 (3)	0.0601 (19)	0.604 (6)
H21	0.2017	1.1158	0.3199	0.090*	0.604 (6)
C65	0.2869 (9)	1.1988 (10)	0.3911 (6)	0.097 (5)	0.604 (6)
H65A	0.3521	1.1737	0.3858	0.146*	0.604 (6)
H65B	0.2634	1.1763	0.4284	0.146*	0.604 (6)
H65C	0.3069	1.2666	0.4013	0.146*	0.604 (6)

### Atomic displacement parameters (Å^2^)

**Table:**

	*U*^11^	*U*^22^	*U*^33^	*U*^12^	*U*^13^	*U*^23^
Mn1	0.0258 (3)	0.0298 (3)	0.0229 (4)	0.0102 (2)	0.0071 (3)	0.0024 (2)
Mn2	0.0272 (3)	0.0296 (3)	0.0235 (4)	0.0094 (2)	0.0063 (3)	0.0016 (2)
Mn3	0.0291 (3)	0.0275 (3)	0.0282 (4)	0.0096 (2)	0.0098 (3)	0.0037 (2)
Mn4	0.0316 (3)	0.0298 (3)	0.0354 (4)	0.0108 (2)	0.0152 (3)	0.0057 (3)
Mn5	0.0330 (3)	0.0314 (3)	0.0312 (4)	0.0130 (2)	0.0107 (3)	0.0066 (3)
Mn6	0.0252 (3)	0.0416 (3)	0.0248 (4)	0.0100 (2)	0.0065 (3)	0.0034 (3)
O1	0.0362 (15)	0.0359 (14)	0.0288 (17)	0.0118 (11)	0.0023 (13)	0.0010 (12)
O2	0.0268 (13)	0.0324 (13)	0.0309 (17)	0.0112 (10)	0.0084 (12)	0.0027 (11)
O3	0.0282 (13)	0.0343 (13)	0.0156 (15)	0.0079 (10)	0.0007 (11)	0.0017 (11)
O4	0.0394 (15)	0.0314 (14)	0.0394 (18)	0.0108 (11)	0.0143 (14)	0.0053 (12)
O5	0.0354 (15)	0.0350 (14)	0.0373 (18)	0.0113 (11)	0.0172 (14)	0.0052 (12)
O6	0.0328 (14)	0.0267 (12)	0.0351 (17)	0.0152 (10)	0.0137 (13)	0.0069 (11)
O7	0.0347 (15)	0.0323 (14)	0.0428 (19)	0.0091 (11)	0.0156 (14)	0.0038 (12)
O8	0.0380 (15)	0.0310 (13)	0.0377 (18)	0.0147 (11)	0.0128 (14)	0.0089 (12)
O9	0.0269 (13)	0.0358 (14)	0.0384 (18)	0.0103 (11)	0.0113 (13)	0.0081 (12)
O10	0.0526 (19)	0.0389 (16)	0.062 (2)	0.0151 (14)	0.0267 (18)	0.0087 (15)
O11	0.0353 (15)	0.0475 (16)	0.0356 (18)	0.0193 (13)	0.0147 (14)	0.0072 (13)
O12	0.0302 (13)	0.0324 (13)	0.0283 (16)	0.0137 (11)	0.0061 (12)	0.0036 (11)
O13	0.0279 (14)	0.0626 (19)	0.0253 (17)	0.0038 (13)	0.0064 (13)	0.0052 (14)
O14	0.0275 (14)	0.0323 (13)	0.0259 (16)	0.0076 (10)	0.0046 (13)	0.0020 (11)
O15	0.0253 (13)	0.0362 (14)	0.0245 (16)	0.0061 (10)	0.0088 (12)	0.0014 (11)
O16	0.0373 (15)	0.0383 (15)	0.0384 (19)	0.0078 (12)	0.0112 (14)	0.0037 (13)
O17	0.062 (2)	0.0409 (16)	0.042 (2)	0.0157 (15)	0.0154 (17)	0.0046 (14)
O18	0.0292 (14)	0.0326 (14)	0.0373 (18)	0.0091 (11)	0.0085 (13)	0.0022 (12)
O19	0.0306 (14)	0.0388 (14)	0.0298 (17)	0.0114 (12)	0.0042 (13)	−0.0015 (12)
N1	0.0289 (16)	0.0344 (16)	0.0221 (19)	0.0082 (13)	0.0060 (14)	0.0053 (13)
N2	0.0323 (17)	0.0267 (15)	0.032 (2)	0.0154 (13)	0.0066 (15)	0.0032 (14)
N3	0.0251 (16)	0.0373 (17)	0.035 (2)	0.0057 (13)	0.0103 (15)	0.0076 (15)
N4	0.0363 (18)	0.0334 (16)	0.026 (2)	0.0175 (13)	0.0083 (15)	0.0040 (14)
N5	0.0250 (16)	0.0370 (17)	0.029 (2)	0.0092 (13)	0.0114 (15)	0.0053 (14)
N6	0.0337 (17)	0.0299 (16)	0.026 (2)	0.0067 (13)	0.0056 (15)	0.0013 (14)
N7	0.048 (2)	0.0325 (17)	0.027 (2)	0.0153 (15)	0.0118 (18)	0.0038 (14)
N8	0.0310 (16)	0.0267 (15)	0.035 (2)	0.0103 (12)	0.0090 (16)	0.0046 (14)
N9	0.044 (2)	0.0353 (17)	0.043 (2)	0.0097 (15)	0.0251 (18)	0.0086 (16)
C1	0.0258 (19)	0.037 (2)	0.031 (3)	0.0075 (15)	0.0067 (18)	0.0101 (17)
C2	0.040 (2)	0.046 (2)	0.026 (3)	0.0094 (19)	0.001 (2)	−0.0006 (19)
C3	0.046 (3)	0.062 (3)	0.028 (3)	0.014 (2)	−0.005 (2)	0.003 (2)
C4	0.039 (2)	0.060 (3)	0.040 (3)	0.022 (2)	0.008 (2)	0.012 (2)
C5	0.033 (2)	0.045 (2)	0.033 (3)	0.0152 (17)	0.011 (2)	0.0107 (19)
C6	0.0271 (19)	0.037 (2)	0.023 (2)	0.0071 (15)	0.0067 (17)	0.0059 (16)
C7	0.0254 (18)	0.0296 (18)	0.032 (2)	0.0093 (14)	0.0100 (18)	0.0101 (16)
C8	0.030 (2)	0.0331 (19)	0.031 (2)	0.0135 (16)	0.0039 (18)	−0.0001 (17)
C9	0.044 (2)	0.033 (2)	0.030 (3)	0.0154 (17)	0.007 (2)	0.0022 (17)
C10	0.051 (3)	0.039 (2)	0.039 (3)	0.023 (2)	0.007 (2)	−0.0007 (19)
C11	0.040 (2)	0.044 (2)	0.037 (3)	0.0252 (19)	0.009 (2)	−0.0028 (19)
C12	0.037 (2)	0.043 (2)	0.030 (3)	0.0160 (17)	0.010 (2)	0.0006 (18)
C13	0.0293 (19)	0.0307 (19)	0.026 (2)	0.0126 (15)	0.0023 (17)	−0.0031 (16)
C14	0.0270 (18)	0.0346 (19)	0.024 (2)	0.0088 (15)	0.0085 (17)	−0.0014 (16)
C21	0.033 (2)	0.0288 (18)	0.026 (2)	0.0086 (15)	0.0018 (18)	0.0044 (16)
C22	0.056 (3)	0.043 (2)	0.034 (3)	0.026 (2)	0.016 (2)	0.0101 (19)
C23	0.072 (3)	0.039 (2)	0.047 (3)	0.028 (2)	0.023 (3)	0.009 (2)
C24	0.088 (4)	0.045 (3)	0.043 (3)	0.037 (3)	0.021 (3)	0.008 (2)
C25	0.087 (4)	0.063 (3)	0.059 (4)	0.051 (3)	0.034 (3)	0.015 (3)
C26	0.067 (3)	0.051 (3)	0.044 (3)	0.033 (2)	0.025 (3)	0.011 (2)
C27	0.044 (2)	0.046 (2)	0.032 (3)	0.0251 (19)	0.013 (2)	0.0092 (19)
C28	0.033 (2)	0.042 (2)	0.027 (2)	0.0175 (17)	0.0075 (19)	0.0033 (18)
C29	0.030 (2)	0.040 (2)	0.031 (3)	0.0105 (16)	0.0093 (19)	0.0042 (17)
C30	0.0232 (19)	0.050 (2)	0.031 (3)	0.0053 (17)	0.0033 (18)	0.0036 (19)
C31	0.028 (2)	0.047 (2)	0.043 (3)	0.0059 (17)	0.013 (2)	0.005 (2)
C32	0.039 (2)	0.044 (2)	0.038 (3)	0.0110 (18)	0.019 (2)	0.0088 (19)
C33	0.033 (2)	0.038 (2)	0.031 (2)	0.0113 (16)	0.0126 (19)	0.0063 (17)
C34	0.0259 (19)	0.0311 (18)	0.032 (2)	0.0073 (15)	0.0084 (18)	0.0032 (16)
C35	0.0279 (19)	0.0274 (17)	0.024 (2)	0.0089 (14)	0.0048 (18)	−0.0015 (15)
C36	0.033 (2)	0.0321 (19)	0.029 (2)	0.0063 (16)	0.0108 (19)	0.0037 (17)
C37	0.065 (4)	0.076 (4)	0.094 (6)	0.013 (3)	0.017 (4)	−0.002 (4)
C38	0.109 (8)	0.063 (5)	0.117 (10)	0.003 (5)	0.056 (7)	0.010 (5)
C38B	0.109 (8)	0.063 (5)	0.117 (10)	0.003 (5)	0.056 (7)	0.010 (5)
C39	0.034 (2)	0.039 (2)	0.027 (2)	0.0091 (17)	0.0086 (19)	0.0024 (17)
C40	0.040 (3)	0.047 (3)	0.065 (4)	0.006 (2)	0.002 (3)	0.004 (2)
C41	0.045 (3)	0.050 (4)	0.079 (6)	0.009 (3)	0.022 (4)	0.008 (3)
C41B	0.045 (3)	0.050 (4)	0.079 (6)	0.009 (3)	0.022 (4)	0.008 (3)
C42	0.042 (2)	0.035 (2)	0.030 (3)	0.0137 (17)	0.010 (2)	0.0006 (17)
C43	0.041 (2)	0.033 (2)	0.033 (3)	0.0086 (17)	0.005 (2)	−0.0026 (17)
C44	0.050 (2)	0.030 (2)	0.031 (3)	0.0064 (17)	0.008 (2)	−0.0054 (17)
C45	0.080 (4)	0.038 (2)	0.043 (3)	0.032 (2)	0.021 (3)	0.008 (2)
C46	0.045 (2)	0.036 (2)	0.053 (3)	0.0163 (18)	0.026 (2)	0.016 (2)
C47	0.051 (3)	0.045 (2)	0.068 (4)	0.023 (2)	0.030 (3)	0.031 (2)
C48	0.054 (3)	0.041 (2)	0.059 (4)	0.014 (2)	0.020 (3)	0.026 (2)
C49	0.070 (3)	0.047 (3)	0.082 (4)	0.017 (2)	0.056 (3)	0.017 (3)
N12	0.042 (2)	0.063 (6)	0.032 (2)	0.017 (3)	0.0092 (19)	0.013 (2)
C54	0.048 (3)	0.057 (6)	0.033 (3)	0.007 (2)	0.008 (3)	0.013 (3)
N13	0.061 (3)	0.057 (4)	0.033 (2)	−0.0002 (19)	−0.001 (2)	0.012 (2)
C55	0.048 (4)	0.081 (5)	0.034 (4)	0.026 (3)	−0.001 (3)	0.013 (3)
C56	0.060 (5)	0.066 (5)	0.049 (5)	0.009 (4)	−0.014 (4)	0.015 (4)
C57	0.106 (11)	0.062 (8)	0.032 (6)	−0.002 (7)	−0.007 (7)	0.010 (5)
O25	0.042 (2)	0.063 (6)	0.032 (2)	0.017 (3)	0.0092 (19)	0.013 (2)
C54B	0.048 (3)	0.057 (6)	0.033 (3)	0.007 (2)	0.008 (3)	0.013 (3)
N13B	0.061 (3)	0.057 (4)	0.033 (2)	−0.0002 (19)	−0.001 (2)	0.012 (2)
C56B	0.051 (9)	0.064 (10)	0.030 (9)	−0.005 (8)	−0.007 (8)	0.004 (8)
C57B	0.068 (12)	0.043 (10)	0.042 (14)	0.019 (9)	0.004 (10)	−0.003 (11)
N14	0.0327 (17)	0.0396 (17)	0.029 (2)	0.0151 (14)	0.0115 (16)	0.0069 (15)
C58	0.031 (2)	0.040 (2)	0.021 (2)	0.0088 (16)	0.0023 (18)	−0.0015 (16)
N15	0.0272 (17)	0.0452 (19)	0.032 (2)	0.0108 (14)	0.0048 (16)	0.0024 (15)
C59	0.033 (2)	0.048 (2)	0.034 (3)	0.0161 (18)	0.010 (2)	0.0041 (19)
C60	0.041 (2)	0.056 (3)	0.030 (3)	0.015 (2)	0.013 (2)	0.005 (2)
C61	0.034 (2)	0.052 (3)	0.030 (3)	0.0087 (19)	0.001 (2)	−0.003 (2)
O20	0.0372 (16)	0.0440 (16)	0.0338 (19)	0.0003 (12)	0.0084 (14)	0.0005 (13)
C62	0.046 (2)	0.034 (2)	0.023 (2)	0.0067 (17)	0.003 (2)	−0.0067 (17)
N16	0.0385 (19)	0.0399 (18)	0.034 (2)	0.0102 (15)	0.0074 (18)	−0.0017 (16)
C63	0.055 (3)	0.050 (3)	0.046 (3)	0.013 (2)	0.019 (3)	0.005 (2)
C64	0.041 (3)	0.061 (3)	0.055 (4)	0.014 (2)	0.013 (3)	−0.006 (2)
C15	0.032 (2)	0.034 (2)	0.035 (4)	0.0094 (17)	0.006 (2)	0.0084 (19)
C16	0.026 (3)	0.043 (2)	0.041 (7)	0.010 (2)	0.003 (4)	0.012 (3)
C17	0.030 (3)	0.052 (3)	0.074 (8)	0.005 (2)	0.015 (5)	0.016 (4)
C18	0.041 (3)	0.040 (3)	0.073 (7)	0.004 (2)	0.018 (5)	0.015 (4)
C19	0.037 (3)	0.033 (2)	0.054 (7)	0.008 (2)	0.012 (4)	0.012 (3)
C20	0.025 (2)	0.032 (2)	0.041 (4)	0.0091 (16)	0.006 (2)	0.011 (2)
O22	0.081 (4)	0.079 (4)	0.057 (4)	−0.009 (3)	0.014 (3)	0.026 (3)
C69	0.079 (8)	0.054 (5)	0.046 (6)	−0.007 (5)	0.012 (5)	0.021 (4)
C15B	0.032 (2)	0.034 (2)	0.035 (4)	0.0094 (17)	0.006 (2)	0.0084 (19)
C16B	0.026 (3)	0.043 (2)	0.041 (7)	0.010 (2)	0.003 (4)	0.012 (3)
C17B	0.030 (3)	0.052 (3)	0.074 (8)	0.005 (2)	0.015 (5)	0.016 (4)
C18B	0.041 (3)	0.040 (3)	0.073 (7)	0.004 (2)	0.018 (5)	0.015 (4)
C19B	0.037 (3)	0.033 (2)	0.054 (7)	0.008 (2)	0.012 (4)	0.012 (3)
C20B	0.025 (2)	0.032 (2)	0.041 (4)	0.0091 (16)	0.006 (2)	0.011 (2)
O23	0.120 (14)	0.108 (13)	0.082 (13)	0.075 (11)	0.054 (11)	0.046 (10)
C67	0.14 (2)	0.14 (2)	0.10 (2)	0.035 (17)	0.053 (17)	0.022 (18)
N17	0.130 (16)	0.111 (16)	0.089 (14)	0.059 (12)	0.055 (13)	0.028 (12)
C66	0.13 (2)	0.12 (2)	0.085 (18)	0.064 (16)	0.053 (16)	0.033 (15)
C68	0.125 (15)	0.107 (15)	0.086 (14)	0.062 (12)	0.052 (12)	0.037 (12)
N10	0.029 (3)	0.0409 (18)	0.035 (5)	0.012 (3)	0.005 (3)	0.004 (4)
C50	0.027 (5)	0.033 (5)	0.035 (6)	0.009 (4)	0.007 (4)	0.002 (5)
N11	0.033 (4)	0.036 (5)	0.033 (6)	0.003 (4)	0.012 (4)	0.005 (4)
C51	0.021 (4)	0.047 (4)	0.033 (4)	0.005 (3)	0.007 (3)	0.006 (3)
C52	0.028 (5)	0.047 (5)	0.035 (5)	0.007 (4)	0.013 (4)	0.010 (4)
C53	0.066 (10)	0.056 (7)	0.042 (9)	0.009 (8)	0.039 (7)	−0.005 (7)
O24	0.029 (3)	0.0409 (18)	0.035 (5)	0.012 (3)	0.005 (3)	0.004 (4)
C50B	0.034 (5)	0.044 (5)	0.039 (7)	0.012 (4)	0.010 (5)	0.010 (6)
N11B	0.041 (5)	0.044 (7)	0.033 (6)	−0.001 (5)	0.011 (5)	0.000 (5)
C52B	0.042 (6)	0.074 (9)	0.048 (7)	−0.012 (7)	0.014 (5)	0.015 (7)
C53B	0.047 (7)	0.059 (8)	0.039 (9)	−0.002 (6)	0.034 (7)	−0.007 (7)
O21	0.081 (4)	0.042 (3)	0.057 (4)	0.008 (3)	0.023 (4)	0.004 (3)
C65	0.070 (7)	0.140 (11)	0.055 (8)	−0.038 (7)	0.016 (6)	0.002 (7)

### Geometric parameters (Å, º)

**Table:**

Mn1—O3	2.218 (3)	C40—H40A	0.9900
Mn1—O15	2.225 (2)	C40—H40B	0.9900
Mn1—O9	2.228 (3)	C40—H40C	0.9900
Mn1—O16	2.244 (3)	C40—H40D	0.9900
Mn1—O6	2.245 (2)	C41—H41A	0.9800
Mn1—O18	2.251 (3)	C41—H41B	0.9800
Mn1—O12	2.281 (3)	C41—H41C	0.9800
Mn2—O1	1.843 (3)	C41B—H41D	0.9800
Mn2—O15	1.891 (3)	C41B—H41E	0.9800
Mn2—N1	1.971 (3)	C41B—H41F	0.9800
Mn2—N6	2.044 (3)	C42—H42	0.9500
Mn2—O14	2.154 (2)	C43—C44	1.361 (6)
Mn3—O4	1.856 (3)	C43—H43	0.9500
Mn3—O3	1.903 (2)	C44—H44	0.9500
Mn3—N2	2.000 (3)	C45—H45A	0.9800
Mn3—N8	2.061 (3)	C45—H45B	0.9800
Mn3—O2	2.163 (3)	C45—H45C	0.9800
Mn3—O19	2.212 (3)	C46—H46	0.9500
Mn4—O7	1.874 (3)	C47—C48	1.353 (6)
Mn4—O6	1.908 (3)	C47—H47	0.9500
Mn4—O5	1.962 (3)	C48—H48	0.9500
Mn4—N3	1.975 (3)	C49—H49A	0.9800
Mn4—O24	2.118 (14)	C49—H49B	0.9800
Mn4—O20	2.277 (3)	C49—H49C	0.9800
Mn4—N10	2.342 (19)	N12—C54	1.302 (12)
Mn5—O10	1.854 (3)	N12—C55	1.378 (17)
Mn5—O9	1.940 (3)	C54—N13	1.336 (12)
Mn5—N4	1.941 (3)	C54—H54	0.9500
Mn5—O8	1.948 (3)	N13—C56	1.390 (15)
Mn5—O25	2.23 (3)	N13—C57	1.459 (12)
Mn5—N12	2.238 (18)	C55—C56	1.330 (11)
Mn5—O17	2.248 (4)	C55—H55	0.9500
Mn6—O13	1.855 (3)	C56—H56A	0.9500
Mn6—O12	1.907 (3)	C57—H57A	0.9800
Mn6—N5	1.973 (3)	C57—H57B	0.9800
Mn6—N14	2.039 (4)	C57—H57C	0.9800
Mn6—O11	2.155 (3)	O25—C54B	1.225 (18)
Mn6—O18	2.414 (2)	C54B—N13B	1.318 (17)
O1—C1	1.346 (5)	C54B—H54B	0.9500
O2—C7	1.271 (4)	N13B—C57B	1.463 (17)
O3—N1	1.404 (4)	N13B—C56B	1.468 (19)
O4—C8	1.338 (4)	C56B—H56B	0.9800
O5—C14	1.299 (5)	C56B—H56C	0.9800
O6—N2	1.414 (4)	C56B—H56D	0.9800
O7—C15B	1.320 (15)	C57B—H57D	0.9800
O7—C15	1.342 (6)	C57B—H57E	0.9800
O8—C21	1.294 (4)	C57B—H57F	0.9800
O9—N3	1.414 (4)	N14—C58	1.326 (5)
O10—C22	1.330 (5)	N14—C59	1.378 (5)
O11—C28	1.276 (5)	C58—N15	1.342 (5)
O12—N4	1.401 (4)	C58—H58	0.9500
O13—C29	1.334 (5)	N15—C60	1.388 (5)
O14—C35	1.275 (5)	N15—C61	1.463 (5)
O15—N5	1.406 (4)	C59—C60	1.363 (6)
O16—C36	1.267 (5)	C59—H59	0.9500
O17—C36	1.260 (5)	C60—H60	0.9500
O18—C39	1.267 (5)	C61—H61A	0.9800
O19—C39	1.248 (5)	C61—H61B	0.9800
N1—C7	1.333 (5)	C61—H61C	0.9800
N2—C14	1.323 (4)	O20—C62	1.242 (5)
N3—C21	1.310 (5)	C62—N16	1.318 (5)
N4—C28	1.326 (5)	C62—H62	0.9500
N5—C35	1.329 (5)	N16—C64	1.456 (5)
N6—C42	1.327 (5)	N16—C63	1.457 (6)
N6—C43	1.374 (5)	C63—H63A	0.9800
N7—C42	1.332 (5)	C63—H63B	0.9800
N7—C44	1.366 (6)	C63—H63C	0.9800
N7—C45	1.464 (5)	C64—H64A	0.9800
N8—C46	1.320 (5)	C64—H64B	0.9800
N8—C47	1.369 (5)	C64—H64C	0.9800
N9—C46	1.332 (5)	C15—C16	1.410 (7)
N9—C48	1.361 (6)	C15—C20	1.423 (7)
N9—C49	1.465 (5)	C16—C17	1.374 (8)
C1—C2	1.385 (6)	C16—H16	0.9500
C1—C6	1.415 (5)	C17—C18	1.399 (8)
C2—C3	1.385 (6)	C17—H17	0.9500
C2—H2	0.9500	C18—C19	1.375 (8)
C3—C4	1.394 (7)	C18—H18	0.9500
C3—H3	0.9500	C19—C20	1.396 (7)
C4—C5	1.372 (7)	C19—H19	0.9500
C4—H4	0.9500	O22—C69	1.405 (12)
C5—C6	1.401 (6)	O22—H22	0.8400
C5—H5	0.9500	C69—H69A	0.9800
C6—C7	1.464 (6)	C69—H69B	0.9800
C8—C9	1.409 (5)	C69—H69C	0.9800
C8—C13	1.410 (6)	C15B—C16B	1.398 (16)
C9—C10	1.384 (6)	C15B—C20B	1.411 (15)
C9—H9	0.9500	C16B—C17B	1.365 (17)
C10—C11	1.386 (7)	C16B—H16B	0.9500
C10—H10	0.9500	C17B—C18B	1.398 (17)
C11—C12	1.379 (6)	C17B—H17B	0.9500
C11—H11	0.9500	C18B—C19B	1.374 (17)
C12—C13	1.414 (5)	C18B—H18B	0.9500
C12—H12	0.9500	C19B—C20B	1.385 (16)
C13—C14	1.466 (5)	C19B—H19B	0.9500
C21—C20B	1.449 (15)	O23—C68	1.16 (3)
C21—C20	1.470 (7)	C67—N17	1.51 (6)
C22—C23	1.398 (6)	C67—H67A	0.9800
C22—C27	1.412 (6)	C67—H67B	0.9800
C23—C24	1.392 (7)	C67—H67C	0.9800
C23—H23	0.9500	N17—C66	1.39 (7)
C24—C25	1.389 (8)	N17—C68	1.45 (7)
C24—H24	0.9500	C66—H66A	0.9800
C25—C26	1.381 (7)	C66—H66B	0.9800
C25—H25	0.9500	C66—H66C	0.9800
C26—C27	1.400 (6)	C68—H68	0.9500
C26—H26	0.9500	N10—C50	1.306 (15)
C27—C28	1.479 (6)	N10—C51	1.377 (17)
C29—C30	1.400 (5)	C50—N11	1.341 (14)
C29—C34	1.422 (6)	C50—H50	0.9500
C30—C31	1.386 (6)	N11—C52	1.382 (14)
C30—H30	0.9500	N11—C53	1.468 (14)
C31—C32	1.383 (6)	C51—C52	1.360 (12)
C31—H31	0.9500	C51—H51	0.9500
C32—C33	1.381 (5)	C52—H52	0.9500
C32—H32	0.9500	C53—H53A	0.9800
C33—C34	1.394 (6)	C53—H53B	0.9800
C33—H33	0.9500	C53—H53C	0.9800
C34—C35	1.476 (5)	O24—C50B	1.232 (14)
C36—C37	1.480 (8)	C50B—N11B	1.319 (14)
C37—C38	1.484 (12)	C50B—H50B	0.9500
C37—C38B	1.697 (18)	N11B—C52B	1.470 (14)
C37—H37A	0.9900	N11B—C53B	1.473 (15)
C37—H37B	0.9900	C52B—H52A	0.9800
C37—H37C	0.9900	C52B—H52B	0.9800
C37—H37D	0.9900	C52B—H52C	0.9800
C38—H38A	0.9800	C53B—H53D	0.9800
C38—H38B	0.9800	C53B—H53E	0.9800
C38—H38C	0.9800	C53B—H53F	0.9800
C38B—H38D	0.9800	O21—C65	1.297 (12)
C38B—H38E	0.9800	O21—H21	0.8400
C38B—H38F	0.9800	C65—H65A	0.9800
C39—C40	1.522 (6)	C65—H65B	0.9800
C40—C41	1.485 (8)	C65—H65C	0.9800
C40—C41B	1.62 (2)		
			
O3—Mn1—O15	75.85 (9)	C36—C37—H37D	110.0
O3—Mn1—O9	153.71 (9)	C38B—C37—H37D	110.0
O15—Mn1—O9	124.49 (10)	H37C—C37—H37D	108.4
O3—Mn1—O16	84.18 (10)	C37—C38—H38A	109.5
O15—Mn1—O16	69.48 (10)	C37—C38—H38B	109.5
O9—Mn1—O16	88.10 (11)	H38A—C38—H38B	109.5
O3—Mn1—O6	78.37 (9)	C37—C38—H38C	109.5
O15—Mn1—O6	151.07 (10)	H38A—C38—H38C	109.5
O9—Mn1—O6	77.34 (9)	H38B—C38—H38C	109.5
O16—Mn1—O6	95.15 (10)	C37—C38B—H38D	109.5
O3—Mn1—O18	80.57 (10)	C37—C38B—H38E	109.5
O15—Mn1—O18	89.92 (10)	H38D—C38B—H38E	109.5
O9—Mn1—O18	112.94 (11)	C37—C38B—H38F	109.5
O16—Mn1—O18	156.90 (11)	H38D—C38B—H38F	109.5
O6—Mn1—O18	98.64 (9)	H38E—C38B—H38F	109.5
O3—Mn1—O12	134.10 (9)	O19—C39—O18	125.3 (4)
O15—Mn1—O12	74.00 (10)	O19—C39—C40	118.5 (4)
O9—Mn1—O12	71.61 (9)	O18—C39—C40	116.2 (4)
O16—Mn1—O12	115.64 (10)	C41—C40—C39	117.0 (5)
O6—Mn1—O12	134.62 (10)	C39—C40—C41B	107.9 (9)
O18—Mn1—O12	65.70 (9)	C41—C40—H40A	108.0
O1—Mn2—O15	176.08 (11)	C39—C40—H40A	108.0
O1—Mn2—N1	89.39 (13)	C41—C40—H40B	108.0
O15—Mn2—N1	94.04 (12)	C39—C40—H40B	108.0
O1—Mn2—N6	87.19 (13)	H40A—C40—H40B	107.3
O15—Mn2—N6	89.00 (13)	C39—C40—H40C	110.1
N1—Mn2—N6	164.16 (13)	C41B—C40—H40C	110.1
O1—Mn2—O14	103.80 (12)	C39—C40—H40D	110.1
O15—Mn2—O14	77.57 (10)	C41B—C40—H40D	110.1
N1—Mn2—O14	98.70 (11)	H40C—C40—H40D	108.4
N6—Mn2—O14	97.14 (11)	C40—C41—H41A	109.5
O4—Mn3—O3	175.89 (13)	C40—C41—H41B	109.5
O4—Mn3—N2	89.08 (12)	H41A—C41—H41B	109.5
O3—Mn3—N2	91.15 (11)	C40—C41—H41C	109.5
O4—Mn3—N8	90.49 (12)	H41A—C41—H41C	109.5
O3—Mn3—N8	89.60 (11)	H41B—C41—H41C	109.5
N2—Mn3—N8	175.57 (14)	C40—C41B—H41D	109.5
O4—Mn3—O2	98.36 (12)	C40—C41B—H41E	109.5
O3—Mn3—O2	77.54 (10)	H41D—C41B—H41E	109.5
N2—Mn3—O2	98.18 (12)	C40—C41B—H41F	109.5
N8—Mn3—O2	86.25 (12)	H41D—C41B—H41F	109.5
O4—Mn3—O19	97.27 (12)	H41E—C41B—H41F	109.5
O3—Mn3—O19	86.84 (10)	N6—C42—N7	110.7 (4)
N2—Mn3—O19	90.31 (12)	N6—C42—H42	124.7
N8—Mn3—O19	85.37 (12)	N7—C42—H42	124.7
O2—Mn3—O19	162.30 (10)	C44—C43—N6	108.6 (4)
O7—Mn4—O6	178.22 (13)	C44—C43—H43	125.7
O7—Mn4—O5	98.85 (11)	N6—C43—H43	125.7
O6—Mn4—O5	81.59 (10)	C43—C44—N7	106.5 (4)
O7—Mn4—N3	89.37 (12)	C43—C44—H44	126.8
O6—Mn4—N3	90.12 (11)	N7—C44—H44	126.8
O5—Mn4—N3	171.42 (12)	N7—C45—H45A	109.5
O7—Mn4—O24	88.3 (5)	N7—C45—H45B	109.5
O6—Mn4—O24	93.5 (5)	H45A—C45—H45B	109.5
O5—Mn4—O24	90.2 (10)	N7—C45—H45C	109.5
N3—Mn4—O24	92.5 (10)	H45A—C45—H45C	109.5
O7—Mn4—O20	87.11 (12)	H45B—C45—H45C	109.5
O6—Mn4—O20	91.20 (11)	N8—C46—N9	111.2 (4)
O5—Mn4—O20	87.30 (12)	N8—C46—H46	124.4
N3—Mn4—O20	90.67 (13)	N9—C46—H46	124.4
O24—Mn4—O20	174.4 (6)	C48—C47—N8	109.5 (4)
O7—Mn4—N10	90.6 (6)	C48—C47—H47	125.3
O6—Mn4—N10	91.2 (6)	N8—C47—H47	125.3
O5—Mn4—N10	88.0 (12)	C47—C48—N9	106.2 (4)
N3—Mn4—N10	94.5 (12)	C47—C48—H48	126.9
O20—Mn4—N10	174.3 (10)	N9—C48—H48	126.9
O10—Mn5—O9	175.19 (15)	N9—C49—H49A	109.5
O10—Mn5—N4	89.52 (13)	N9—C49—H49B	109.5
O9—Mn5—N4	92.24 (12)	H49A—C49—H49B	109.5
O10—Mn5—O8	96.51 (12)	N9—C49—H49C	109.5
O9—Mn5—O8	81.71 (10)	H49A—C49—H49C	109.5
N4—Mn5—O8	173.95 (12)	H49B—C49—H49C	109.5
O10—Mn5—O25	90.8 (12)	C54—N12—C55	105.3 (13)
O9—Mn5—O25	93.7 (12)	C54—N12—Mn5	126.7 (12)
N4—Mn5—O25	89.6 (18)	C55—N12—Mn5	127.6 (12)
O8—Mn5—O25	90.8 (17)	N12—C54—N13	112.2 (12)
O10—Mn5—N12	94.8 (7)	N12—C54—H54	123.9
O9—Mn5—N12	89.7 (7)	N13—C54—H54	123.9
N4—Mn5—N12	89.9 (10)	C54—N13—C56	106.0 (10)
O8—Mn5—N12	90.1 (10)	C54—N13—C57	126.2 (12)
O10—Mn5—O17	86.23 (14)	C56—N13—C57	127.4 (16)
O9—Mn5—O17	89.16 (12)	C56—C55—N12	109.9 (10)
N4—Mn5—O17	94.78 (13)	C56—C55—H55	125.1
O8—Mn5—O17	85.14 (12)	N12—C55—H55	125.1
O25—Mn5—O17	175 (2)	C55—C56—N13	106.3 (9)
N12—Mn5—O17	175.2 (9)	C55—C56—H56A	126.8
O13—Mn6—O12	172.90 (12)	N13—C56—H56A	126.8
O13—Mn6—N5	87.92 (13)	N13—C57—H57A	109.5
O12—Mn6—N5	93.24 (12)	N13—C57—H57B	109.5
O13—Mn6—N14	86.67 (13)	H57A—C57—H57B	109.5
O12—Mn6—N14	91.47 (12)	N13—C57—H57C	109.5
N5—Mn6—N14	172.31 (13)	H57A—C57—H57C	109.5
O13—Mn6—O11	109.23 (12)	H57B—C57—H57C	109.5
O12—Mn6—O11	77.70 (10)	C54B—O25—Mn5	124 (2)
N5—Mn6—O11	94.14 (12)	O25—C54B—N13B	124 (3)
N14—Mn6—O11	92.80 (12)	O25—C54B—H54B	117.9
O13—Mn6—O18	105.00 (12)	N13B—C54B—H54B	117.9
O12—Mn6—O18	68.20 (9)	C54B—N13B—C57B	122 (2)
N5—Mn6—O18	83.69 (11)	C54B—N13B—C56B	120 (2)
N14—Mn6—O18	92.44 (11)	C57B—N13B—C56B	117 (2)
O11—Mn6—O18	145.60 (10)	N13B—C56B—H56B	109.5
C1—O1—Mn2	132.0 (3)	N13B—C56B—H56C	109.5
C7—O2—Mn3	108.5 (2)	H56B—C56B—H56C	109.5
N1—O3—Mn3	115.6 (2)	N13B—C56B—H56D	109.5
N1—O3—Mn1	116.35 (19)	H56B—C56B—H56D	109.5
Mn3—O3—Mn1	119.92 (12)	H56C—C56B—H56D	109.5
C8—O4—Mn3	129.4 (2)	N13B—C57B—H57D	109.5
C14—O5—Mn4	111.5 (2)	N13B—C57B—H57E	109.5
N2—O6—Mn4	113.01 (19)	H57D—C57B—H57E	109.5
N2—O6—Mn1	122.9 (2)	N13B—C57B—H57F	109.5
Mn4—O6—Mn1	123.29 (12)	H57D—C57B—H57F	109.5
C15B—O7—Mn4	120.4 (14)	H57E—C57B—H57F	109.5
C15—O7—Mn4	125.1 (5)	C58—N14—C59	106.4 (4)
C21—O8—Mn5	112.1 (2)	C58—N14—Mn6	127.1 (3)
N3—O9—Mn5	111.1 (2)	C59—N14—Mn6	126.6 (3)
N3—O9—Mn1	117.9 (2)	N14—C58—N15	110.9 (3)
Mn5—O9—Mn1	111.83 (12)	N14—C58—H58	124.5
C22—O10—Mn5	131.5 (3)	N15—C58—H58	124.5
C28—O11—Mn6	110.0 (2)	C58—N15—C60	107.6 (3)
N4—O12—Mn6	117.1 (2)	C58—N15—C61	126.2 (3)
N4—O12—Mn1	114.1 (2)	C60—N15—C61	126.2 (4)
Mn6—O12—Mn1	106.68 (12)	C60—C59—N14	109.3 (4)
C29—O13—Mn6	131.9 (3)	C60—C59—H59	125.3
C35—O14—Mn2	110.0 (2)	N14—C59—H59	125.3
N5—O15—Mn2	118.4 (2)	C59—C60—N15	105.8 (4)
N5—O15—Mn1	113.1 (2)	C59—C60—H60	127.1
Mn2—O15—Mn1	110.61 (11)	N15—C60—H60	127.1
C36—O16—Mn1	127.3 (3)	N15—C61—H61A	109.5
C36—O17—Mn5	136.0 (3)	N15—C61—H61B	109.5
C39—O18—Mn1	130.7 (2)	H61A—C61—H61B	109.5
C39—O18—Mn6	132.4 (2)	N15—C61—H61C	109.5
Mn1—O18—Mn6	92.30 (9)	H61A—C61—H61C	109.5
C39—O19—Mn3	134.6 (3)	H61B—C61—H61C	109.5
C7—N1—O3	113.6 (3)	C62—O20—Mn4	124.6 (3)
C7—N1—Mn2	131.5 (3)	O20—C62—N16	123.8 (4)
O3—N1—Mn2	113.5 (2)	O20—C62—H62	118.1
C14—N2—O6	112.2 (3)	N16—C62—H62	118.1
C14—N2—Mn3	129.9 (3)	C62—N16—C64	121.8 (4)
O6—N2—Mn3	117.9 (2)	C62—N16—C63	121.5 (4)
C21—N3—O9	113.6 (3)	C64—N16—C63	116.6 (4)
C21—N3—Mn4	128.4 (3)	N16—C63—H63A	109.5
O9—N3—Mn4	118.0 (2)	N16—C63—H63B	109.5
C28—N4—O12	113.9 (3)	H63A—C63—H63B	109.5
C28—N4—Mn5	133.5 (3)	N16—C63—H63C	109.5
O12—N4—Mn5	112.5 (2)	H63A—C63—H63C	109.5
C35—N5—O15	112.7 (3)	H63B—C63—H63C	109.5
C35—N5—Mn6	133.7 (3)	N16—C64—H64A	109.5
O15—N5—Mn6	113.5 (2)	N16—C64—H64B	109.5
C42—N6—C43	106.3 (3)	H64A—C64—H64B	109.5
C42—N6—Mn2	125.5 (3)	N16—C64—H64C	109.5
C43—N6—Mn2	128.1 (3)	H64A—C64—H64C	109.5
C42—N7—C44	107.9 (3)	H64B—C64—H64C	109.5
C42—N7—C45	125.6 (4)	O7—C15—C16	118.4 (7)
C44—N7—C45	126.4 (4)	O7—C15—C20	123.4 (8)
C46—N8—C47	105.6 (3)	C16—C15—C20	118.2 (5)
C46—N8—Mn3	124.4 (3)	C17—C16—C15	121.5 (6)
C47—N8—Mn3	129.9 (3)	C17—C16—H16	119.2
C46—N9—C48	107.6 (3)	C15—C16—H16	119.2
C46—N9—C49	126.7 (4)	C16—C17—C18	120.1 (6)
C48—N9—C49	125.7 (4)	C16—C17—H17	119.9
O1—C1—C2	117.0 (4)	C18—C17—H17	119.9
O1—C1—C6	123.4 (4)	C19—C18—C17	119.3 (6)
C2—C1—C6	119.6 (4)	C19—C18—H18	120.4
C3—C2—C1	120.8 (4)	C17—C18—H18	120.4
C3—C2—H2	119.6	C18—C19—C20	122.2 (6)
C1—C2—H2	119.6	C18—C19—H19	118.9
C2—C3—C4	120.4 (5)	C20—C19—H19	118.9
C2—C3—H3	119.8	C19—C20—C15	118.7 (5)
C4—C3—H3	119.8	C19—C20—C21	119.4 (7)
C5—C4—C3	119.0 (4)	C15—C20—C21	121.8 (8)
C5—C4—H4	120.5	C69—O22—H22	109.5
C3—C4—H4	120.5	O22—C69—H69A	109.5
C4—C5—C6	122.1 (4)	O22—C69—H69B	109.5
C4—C5—H5	118.9	H69A—C69—H69B	109.5
C6—C5—H5	118.9	O22—C69—H69C	109.5
C5—C6—C1	118.1 (4)	H69A—C69—H69C	109.5
C5—C6—C7	118.1 (3)	H69B—C69—H69C	109.5
C1—C6—C7	123.7 (3)	O7—C15B—C16B	120 (2)
O2—C7—N1	120.8 (4)	O7—C15B—C20B	122 (3)
O2—C7—C6	120.6 (3)	C16B—C15B—C20B	118.3 (15)
N1—C7—C6	118.6 (3)	C17B—C16B—C15B	120.7 (18)
O4—C8—C9	116.9 (4)	C17B—C16B—H16B	119.7
O4—C8—C13	123.8 (3)	C15B—C16B—H16B	119.7
C9—C8—C13	119.2 (3)	C16B—C17B—C18B	120.7 (18)
C10—C9—C8	120.1 (4)	C16B—C17B—H17B	119.7
C10—C9—H9	120.0	C18B—C17B—H17B	119.7
C8—C9—H9	120.0	C19B—C18B—C17B	119.6 (17)
C9—C10—C11	121.2 (4)	C19B—C18B—H18B	120.2
C9—C10—H10	119.4	C17B—C18B—H18B	120.2
C11—C10—H10	119.4	C18B—C19B—C20B	120.4 (17)
C12—C11—C10	119.4 (4)	C18B—C19B—H19B	119.8
C12—C11—H11	120.3	C20B—C19B—H19B	119.8
C10—C11—H11	120.3	C19B—C20B—C15B	120.3 (15)
C11—C12—C13	121.2 (4)	C19B—C20B—C21	115 (2)
C11—C12—H12	119.4	C15B—C20B—C21	125 (2)
C13—C12—H12	119.4	N17—C67—H67A	109.5
C8—C13—C12	118.8 (3)	N17—C67—H67B	109.5
C8—C13—C14	123.6 (3)	H67A—C67—H67B	109.5
C12—C13—C14	117.6 (4)	N17—C67—H67C	109.5
O5—C14—N2	120.7 (3)	H67A—C67—H67C	109.5
O5—C14—C13	119.5 (3)	H67B—C67—H67C	109.5
N2—C14—C13	119.7 (3)	C66—N17—C68	126 (5)
O8—C21—N3	120.7 (3)	C66—N17—C67	123 (5)
O8—C21—C20B	122.7 (13)	C68—N17—C67	110 (4)
N3—C21—C20B	116.3 (12)	N17—C66—H66A	109.5
O8—C21—C20	118.3 (5)	N17—C66—H66B	109.5
N3—C21—C20	121.0 (5)	H66A—C66—H66B	109.5
O10—C22—C23	116.9 (4)	N17—C66—H66C	109.5
O10—C22—C27	123.9 (4)	H66A—C66—H66C	109.5
C23—C22—C27	119.2 (4)	H66B—C66—H66C	109.5
C24—C23—C22	120.7 (5)	O23—C68—N17	129 (4)
C24—C23—H23	119.7	O23—C68—H68	115.7
C22—C23—H23	119.7	N17—C68—H68	115.7
C25—C24—C23	120.2 (4)	C50—N10—C51	105.3 (14)
C25—C24—H24	119.9	C50—N10—Mn4	125.4 (14)
C23—C24—H24	119.9	C51—N10—Mn4	128.9 (14)
C26—C25—C24	119.5 (4)	N10—C50—N11	112.0 (14)
C26—C25—H25	120.3	N10—C50—H50	124.0
C24—C25—H25	120.3	N11—C50—H50	124.0
C25—C26—C27	121.6 (5)	C50—N11—C52	107.4 (12)
C25—C26—H26	119.2	C50—N11—C53	122.2 (15)
C27—C26—H26	119.2	C52—N11—C53	130.0 (15)
C26—C27—C22	118.9 (4)	C52—C51—N10	110.3 (11)
C26—C27—C28	117.6 (4)	C52—C51—H51	124.8
C22—C27—C28	123.6 (3)	N10—C51—H51	124.8
O11—C28—N4	120.7 (4)	C51—C52—N11	104.9 (10)
O11—C28—C27	121.4 (3)	C51—C52—H52	127.5
N4—C28—C27	117.8 (4)	N11—C52—H52	127.5
O13—C29—C30	118.0 (4)	N11—C53—H53A	109.5
O13—C29—C34	124.0 (3)	N11—C53—H53B	109.5
C30—C29—C34	118.0 (4)	H53A—C53—H53B	109.5
C31—C30—C29	121.8 (4)	N11—C53—H53C	109.5
C31—C30—H30	119.1	H53A—C53—H53C	109.5
C29—C30—H30	119.1	H53B—C53—H53C	109.5
C32—C31—C30	119.9 (4)	C50B—O24—Mn4	134.1 (14)
C32—C31—H31	120.0	O24—C50B—N11B	122.5 (16)
C30—C31—H31	120.0	O24—C50B—H50B	118.7
C33—C32—C31	119.2 (4)	N11B—C50B—H50B	118.7
C33—C32—H32	120.4	C50B—N11B—C52B	120.7 (13)
C31—C32—H32	120.4	C50B—N11B—C53B	123.0 (15)
C32—C33—C34	122.2 (4)	C52B—N11B—C53B	115.4 (14)
C32—C33—H33	118.9	N11B—C52B—H52A	109.5
C34—C33—H33	118.9	N11B—C52B—H52B	109.5
C33—C34—C29	118.7 (3)	H52A—C52B—H52B	109.5
C33—C34—C35	118.8 (4)	N11B—C52B—H52C	109.5
C29—C34—C35	122.5 (4)	H52A—C52B—H52C	109.5
O14—C35—N5	121.3 (3)	H52B—C52B—H52C	109.5
O14—C35—C34	120.8 (3)	N11B—C53B—H53D	109.5
N5—C35—C34	117.9 (4)	N11B—C53B—H53E	109.5
O17—C36—O16	124.6 (4)	H53D—C53B—H53E	109.5
O17—C36—C37	115.1 (4)	N11B—C53B—H53F	109.5
O16—C36—C37	120.2 (5)	H53D—C53B—H53F	109.5
C36—C37—C38	113.5 (8)	H53E—C53B—H53F	109.5
C36—C37—C38B	108.5 (9)	C65—O21—H21	109.5
C36—C37—H37A	108.9	O21—C65—H65A	109.5
C38—C37—H37A	108.9	O21—C65—H65B	109.5
C36—C37—H37B	108.9	H65A—C65—H65B	109.5
C38—C37—H37B	108.9	O21—C65—H65C	109.5
H37A—C37—H37B	107.7	H65A—C65—H65C	109.5
C36—C37—H37C	110.0	H65B—C65—H65C	109.5
C38B—C37—H37C	110.0		
			
N1—Mn2—O1—C1	−0.7 (3)	C30—C31—C32—C33	−0.2 (6)
N6—Mn2—O1—C1	−165.2 (3)	C31—C32—C33—C34	0.9 (6)
O14—Mn2—O1—C1	98.1 (3)	C32—C33—C34—C29	−0.9 (6)
N2—Mn3—O4—C8	23.8 (4)	C32—C33—C34—C35	−179.4 (3)
N8—Mn3—O4—C8	−160.6 (4)	O13—C29—C34—C33	179.7 (3)
O2—Mn3—O4—C8	−74.3 (4)	C30—C29—C34—C33	0.2 (5)
O19—Mn3—O4—C8	114.0 (3)	O13—C29—C34—C35	−1.8 (6)
O5—Mn4—O7—C15B	−138.2 (15)	C30—C29—C34—C35	178.6 (3)
N3—Mn4—O7—C15B	44.2 (15)	Mn2—O14—C35—N5	−1.8 (4)
O24—Mn4—O7—C15B	−48.3 (18)	Mn2—O14—C35—C34	178.9 (3)
O20—Mn4—O7—C15B	134.9 (15)	O15—N5—C35—O14	−0.6 (5)
N10—Mn4—O7—C15B	−50.2 (19)	Mn6—N5—C35—O14	175.1 (2)
O5—Mn4—O7—C15	−146.5 (5)	O15—N5—C35—C34	178.7 (3)
N3—Mn4—O7—C15	35.9 (6)	Mn6—N5—C35—C34	−5.6 (5)
O24—Mn4—O7—C15	−56.6 (11)	C33—C34—C35—O14	8.4 (5)
O20—Mn4—O7—C15	126.6 (5)	C29—C34—C35—O14	−170.1 (3)
N10—Mn4—O7—C15	−58.5 (13)	C33—C34—C35—N5	−171.0 (3)
N4—Mn5—O10—C22	−1.2 (4)	C29—C34—C35—N5	10.6 (5)
O8—Mn5—O10—C22	179.3 (4)	Mn5—O17—C36—O16	−28.2 (6)
O25—Mn5—O10—C22	88.4 (19)	Mn5—O17—C36—C37	152.5 (4)
N12—Mn5—O10—C22	88.7 (11)	Mn1—O16—C36—O17	7.5 (5)
O17—Mn5—O10—C22	−96.0 (4)	Mn1—O16—C36—C37	−173.2 (4)
N5—Mn6—O13—C29	14.7 (3)	O17—C36—C37—C38	−67.1 (8)
N14—Mn6—O13—C29	−170.8 (4)	O16—C36—C37—C38	113.5 (7)
O11—Mn6—O13—C29	−79.0 (4)	O17—C36—C37—C38B	10.6 (9)
O18—Mn6—O13—C29	97.6 (3)	O16—C36—C37—C38B	−168.7 (7)
N1—Mn2—O15—N5	94.9 (2)	Mn3—O19—C39—O18	25.4 (6)
N6—Mn2—O15—N5	−100.7 (2)	Mn3—O19—C39—C40	−154.5 (3)
O14—Mn2—O15—N5	−3.1 (2)	Mn1—O18—C39—O19	3.2 (6)
N1—Mn2—O15—Mn1	−37.90 (13)	Mn6—O18—C39—O19	−145.7 (3)
N6—Mn2—O15—Mn1	126.53 (13)	Mn1—O18—C39—C40	−176.9 (3)
O14—Mn2—O15—Mn1	−135.93 (13)	Mn6—O18—C39—C40	34.3 (6)
Mn3—O3—N1—C7	16.6 (3)	O19—C39—C40—C41	14.5 (7)
Mn1—O3—N1—C7	−132.1 (2)	O18—C39—C40—C41	−165.5 (5)
Mn3—O3—N1—Mn2	−175.13 (12)	O19—C39—C40—C41B	70.6 (9)
Mn1—O3—N1—Mn2	36.2 (3)	O18—C39—C40—C41B	−109.4 (9)
Mn4—O6—N2—C14	−8.4 (4)	C43—N6—C42—N7	−0.1 (4)
Mn1—O6—N2—C14	−178.5 (2)	Mn2—N6—C42—N7	−178.1 (3)
Mn4—O6—N2—Mn3	169.47 (15)	C44—N7—C42—N6	−0.1 (5)
Mn1—O6—N2—Mn3	−0.7 (4)	C45—N7—C42—N6	179.8 (4)
Mn5—O9—N3—C21	−8.8 (4)	C42—N6—C43—C44	0.3 (5)
Mn1—O9—N3—C21	−139.7 (3)	Mn2—N6—C43—C44	178.2 (3)
Mn5—O9—N3—Mn4	174.49 (16)	N6—C43—C44—N7	−0.3 (5)
Mn1—O9—N3—Mn4	43.6 (3)	C42—N7—C44—C43	0.2 (5)
Mn6—O12—N4—C28	−7.6 (4)	C45—N7—C44—C43	−179.6 (4)
Mn1—O12—N4—C28	−133.3 (3)	C47—N8—C46—N9	−1.0 (5)
Mn6—O12—N4—Mn5	172.78 (14)	Mn3—N8—C46—N9	−177.9 (3)
Mn1—O12—N4—Mn5	47.1 (3)	C48—N9—C46—N8	1.4 (6)
Mn2—O15—N5—C35	3.2 (4)	C49—N9—C46—N8	−177.3 (4)
Mn1—O15—N5—C35	134.9 (2)	C46—N8—C47—C48	0.3 (6)
Mn2—O15—N5—Mn6	−173.41 (13)	Mn3—N8—C47—C48	176.9 (3)
Mn1—O15—N5—Mn6	−41.7 (3)	N8—C47—C48—N9	0.5 (6)
Mn2—O1—C1—C2	−174.2 (3)	C46—N9—C48—C47	−1.1 (6)
Mn2—O1—C1—C6	5.7 (6)	C49—N9—C48—C47	177.6 (5)
O1—C1—C2—C3	179.0 (4)	C55—N12—C54—N13	−6 (4)
C6—C1—C2—C3	−0.8 (6)	Mn5—N12—C54—N13	−180 (4)
C1—C2—C3—C4	0.4 (7)	N12—C54—N13—C56	5 (5)
C2—C3—C4—C5	0.2 (7)	N12—C54—N13—C57	178 (4)
C3—C4—C5—C6	−0.3 (7)	C54—N12—C55—C56	4 (3)
C4—C5—C6—C1	−0.1 (6)	Mn5—N12—C55—C56	178 (2)
C4—C5—C6—C7	−177.7 (4)	N12—C55—C56—N13	−1 (3)
O1—C1—C6—C5	−179.1 (3)	C54—N13—C56—C55	−2 (4)
C2—C1—C6—C5	0.7 (5)	C57—N13—C56—C55	−175 (4)
O1—C1—C6—C7	−1.6 (6)	Mn5—O25—C54B—N13B	172 (9)
C2—C1—C6—C7	178.2 (3)	O25—C54B—N13B—C57B	179 (7)
Mn3—O2—C7—N1	−12.3 (4)	O25—C54B—N13B—C56B	−12 (14)
Mn3—O2—C7—C6	165.6 (3)	C59—N14—C58—N15	1.1 (4)
O3—N1—C7—O2	−1.1 (5)	Mn6—N14—C58—N15	−178.1 (2)
Mn2—N1—C7—O2	−166.7 (3)	N14—C58—N15—C60	−0.9 (4)
O3—N1—C7—C6	−179.1 (3)	N14—C58—N15—C61	−179.1 (4)
Mn2—N1—C7—C6	15.4 (5)	C58—N14—C59—C60	−0.9 (4)
C5—C6—C7—O2	−8.9 (5)	Mn6—N14—C59—C60	178.3 (3)
C1—C6—C7—O2	173.6 (3)	N14—C59—C60—N15	0.4 (5)
C5—C6—C7—N1	169.1 (3)	C58—N15—C60—C59	0.3 (4)
C1—C6—C7—N1	−8.4 (5)	C61—N15—C60—C59	178.5 (4)
Mn3—O4—C8—C9	160.8 (3)	Mn4—O20—C62—N16	177.0 (3)
Mn3—O4—C8—C13	−20.6 (6)	O20—C62—N16—C64	177.3 (4)
O4—C8—C9—C10	177.5 (4)	O20—C62—N16—C63	−1.1 (6)
C13—C8—C9—C10	−1.2 (6)	C15B—O7—C15—C16	92 (13)
C8—C9—C10—C11	1.0 (7)	Mn4—O7—C15—C16	150.3 (5)
C9—C10—C11—C12	−0.8 (7)	C15B—O7—C15—C20	−90 (13)
C10—C11—C12—C13	0.8 (7)	Mn4—O7—C15—C20	−31.9 (9)
O4—C8—C13—C12	−177.4 (4)	O7—C15—C16—C17	178.1 (7)
C9—C8—C13—C12	1.2 (6)	C20—C15—C16—C17	0.2 (10)
O4—C8—C13—C14	0.6 (6)	C15—C16—C17—C18	−1.1 (11)
C9—C8—C13—C14	179.1 (4)	C16—C17—C18—C19	1.6 (12)
C11—C12—C13—C8	−1.0 (6)	C17—C18—C19—C20	−1.1 (12)
C11—C12—C13—C14	−179.1 (4)	C18—C19—C20—C15	0.1 (10)
Mn4—O5—C14—N2	5.6 (5)	C18—C19—C20—C21	−177.7 (7)
Mn4—O5—C14—C13	−175.8 (3)	O7—C15—C20—C19	−177.5 (8)
O6—N2—C14—O5	1.6 (5)	C16—C15—C20—C19	0.3 (9)
Mn3—N2—C14—O5	−175.9 (3)	O7—C15—C20—C21	0.4 (10)
O6—N2—C14—C13	−176.9 (3)	C16—C15—C20—C21	178.1 (7)
Mn3—N2—C14—C13	5.6 (6)	O8—C21—C20—C19	14.3 (9)
C8—C13—C14—O5	−172.3 (4)	N3—C21—C20—C19	−167.4 (6)
C12—C13—C14—O5	5.7 (6)	C20B—C21—C20—C19	−109 (12)
C8—C13—C14—N2	6.2 (6)	O8—C21—C20—C15	−163.5 (5)
C12—C13—C14—N2	−175.8 (4)	N3—C21—C20—C15	14.8 (9)
Mn5—O8—C21—N3	3.9 (5)	C20B—C21—C20—C15	73 (11)
Mn5—O8—C21—C20B	−169.4 (13)	C15—O7—C15B—C16B	−94 (13)
Mn5—O8—C21—C20	−177.7 (5)	Mn4—O7—C15B—C16B	139.9 (18)
O9—N3—C21—O8	3.3 (6)	C15—O7—C15B—C20B	85 (13)
Mn4—N3—C21—O8	179.6 (3)	Mn4—O7—C15B—C20B	−41 (2)
O9—N3—C21—C20B	177.0 (13)	O7—C15B—C16B—C17B	177 (3)
Mn4—N3—C21—C20B	−6.7 (14)	C20B—C15B—C16B—C17B	−2 (4)
O9—N3—C21—C20	−175.0 (5)	C15B—C16B—C17B—C18B	4 (5)
Mn4—N3—C21—C20	1.3 (7)	C16B—C17B—C18B—C19B	−4 (5)
Mn5—O10—C22—C23	−179.0 (4)	C17B—C18B—C19B—C20B	2 (4)
Mn5—O10—C22—C27	0.0 (7)	C18B—C19B—C20B—C15B	0 (3)
O10—C22—C23—C24	179.4 (5)	C18B—C19B—C20B—C21	−179 (3)
C27—C22—C23—C24	0.3 (8)	O7—C15B—C20B—C19B	−179 (2)
C22—C23—C24—C25	−1.8 (8)	C16B—C15B—C20B—C19B	0 (2)
C23—C24—C25—C26	2.1 (9)	O7—C15B—C20B—C21	0 (2)
C24—C25—C26—C27	−0.9 (8)	C16B—C15B—C20B—C21	179 (2)
C25—C26—C27—C22	−0.6 (8)	O8—C21—C20B—C19B	17 (2)
C25—C26—C27—C28	178.3 (5)	N3—C21—C20B—C19B	−156.1 (16)
O10—C22—C27—C26	−178.1 (5)	C20—C21—C20B—C19B	78 (11)
C23—C22—C27—C26	0.9 (7)	O8—C21—C20B—C15B	−161.7 (13)
O10—C22—C27—C28	3.1 (8)	N3—C21—C20B—C15B	25 (2)
C23—C22—C27—C28	−177.9 (4)	C20—C21—C20B—C15B	−101 (12)
Mn6—O11—C28—N4	1.0 (5)	C66—N17—C68—O23	−180 (4)
Mn6—O11—C28—C27	−178.5 (3)	C67—N17—C68—O23	7 (7)
O12—N4—C28—O11	4.0 (6)	C51—N10—C50—N11	−2 (4)
Mn5—N4—C28—O11	−176.6 (3)	Mn4—N10—C50—N11	−175 (3)
O12—N4—C28—C27	−176.5 (3)	N10—C50—N11—C52	1 (3)
Mn5—N4—C28—C27	2.9 (6)	N10—C50—N11—C53	174 (3)
C26—C27—C28—O11	−3.6 (7)	C50—N10—C51—C52	2 (4)
C22—C27—C28—O11	175.2 (4)	Mn4—N10—C51—C52	175 (2)
C26—C27—C28—N4	176.8 (4)	N10—C51—C52—N11	−2 (2)
C22—C27—C28—N4	−4.4 (7)	C50—N11—C52—C51	0.6 (19)
Mn6—O13—C29—C30	165.6 (3)	C53—N11—C52—C51	−172 (2)
Mn6—O13—C29—C34	−13.9 (6)	Mn4—O24—C50B—N11B	−167 (3)
O13—C29—C30—C31	−179.1 (4)	O24—C50B—N11B—C52B	−2 (4)
C34—C29—C30—C31	0.5 (6)	O24—C50B—N11B—C53B	−171 (3)
C29—C30—C31—C32	−0.4 (6)		

### Hydrogen-bond geometry (Å, º)

**Table:**

*D*—H···*A*	*D*—H	H···*A*	*D*···*A*	*D*—H···*A*
O21—H21···O17	0.84	2.12	2.948 (6)	170
O22—H22···O7	0.84	2.26	3.077 (7)	163
O22—H22···O20	0.84	2.37	2.887 (6)	120
